# TRIM59 promotes breast cancer motility by suppressing p62-selective autophagic degradation of PDCD10

**DOI:** 10.1371/journal.pbio.3000051

**Published:** 2018-11-08

**Authors:** Peng Tan, Youqiong Ye, Lian He, Jiansheng Xie, Ji Jing, Guolin Ma, Hongming Pan, Leng Han, Weidong Han, Yubin Zhou

**Affiliations:** 1 Department of Medical Oncology and Biomedical Research Center, Sir Run Run Shaw Hospital, College of Medicine, Zhejiang University, Hangzhou, China; 2 Center for Translational Cancer Research, Institute of Biosciences and Technology, College of Medicine, Texas A&M University, Houston, Texas, United States of America; 3 Department of Biochemistry and Molecular Biology, University of Texas Health Science Center at Houston McGovern Medical School, Houston, Texas, United States of America; 4 Department of Medical Physiology, College of Medicine, Texas A&M University, Temple, Texas, United States of America; Friedrich Miescher Institute, SWITZERLAND

## Abstract

Cancer cells adopt various modes of migration during metastasis. How the ubiquitination machinery contributes to cancer cell motility remains underexplored. Here, we report that tripartite motif (TRIM) 59 is frequently up-regulated in metastatic breast cancer, which is correlated with advanced clinical stages and reduced survival among breast cancer patients. *TRIM59* knockdown (KD) promoted apoptosis and inhibited tumor growth, while TRIM59 overexpression led to the opposite effects. Importantly, we uncovered TRIM59 as a key regulator of cell contractility and adhesion to control the plasticity of metastatic tumor cells. At the molecular level, we identified programmed cell death protein 10 (PDCD10) as a target of TRIM59. TRIM59 stabilized PDCD10 by suppressing RING finger and transmembrane domain-containing protein 1 (RNFT1)-induced lysine 63 (K63) ubiquitination and subsequent phosphotyrosine-independent ligand for the Lck SH2 domain of 62 kDa (p62)-selective autophagic degradation. TRIM59 promoted PDCD10-mediated suppression of Ras homolog family member A (RhoA)-Rho-associated coiled-coil kinase (ROCK) 1 signaling to control the transition between amoeboid and mesenchymal invasiveness. PDCD10 overexpression or administration of a ROCK inhibitor reversed *TRIM59* loss-induced contractile phenotypes, thereby accelerating cell migration, invasion, and tumor formation. These findings establish the rationale for targeting deregulated TRIM59/PDCD10 to treat breast cancer.

## Abstract 2

Bioinformatic analysis and experiments on clinical samples and mouse models of breast cancer unveil a new signaling protein degradation pathway involving TRIM59 and PDCD10 that modulates breast cancer cell growth, survival, and metastasis.

## Author summary

Cancer cell metastasis is the primary cause of mortality in cancer patients. Changes in cell morphology are critical for cancer cell motility and metastasis, and this process requires spatiotemporal control of the turnover or stabilization of signaling components that regulate cell polarity. Autophagy—a highly regulated intracellular degradation process—provides cancer cells with access to energy sources while preventing mutagenic oxidative stress to suppress tumorigenesis. But the mechanisms underlying how autophagy impacts cell metastasis remain mostly unknown. Here, we identify TRIM59, a protein in the E3 ligase family known to target ubiquitinated proteins for proteasomal or autophagic degradation, as a key component of a novel molecular mechanism in human breast cancer. We show that TRIM59 regulates the degradation of PDCD10, which is involved in programmed cell death and is the main factor for driving the pathogenesis of the devastating familial cerebral cavernous malformation (CCM) disease. We find that TRIM59 has an effect in cell shape and contractility by controlling PDCD10 levels. Our results suggest that targeting the TRIM59-PDCD10 interplay could lead to new therapeutic strategies to treat breast cancer and CCM.

## Introduction

**Fig 1:**
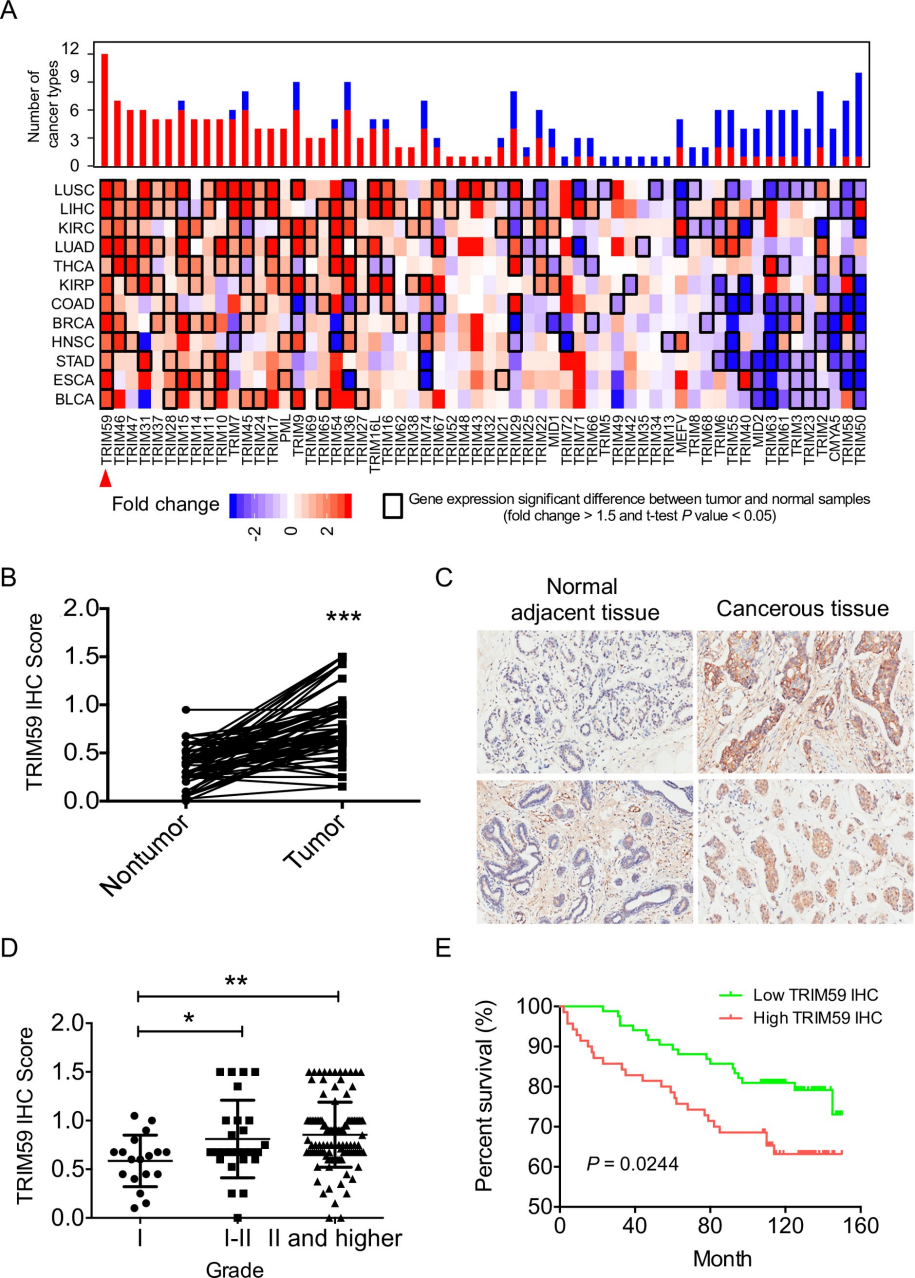
Increased expression of TRIM59 correlates with breast cancer progression and poor survival in patients. (**A**) The fold change of *TRIM* family genes across 12 cancer types (y-axis) compared with their paired controls (obtained from adjacent non-tumorigenic tissues) based on TCGA dataset analysis. The color intensity indicates the range of fold change, and black boxes indicate a significant difference in gene expression between tumor and adjacent non-tumorigenic tissues (fold change > 1.5 and *P* value < 0.05). Upper bars show the frequency of cancer types, with up-regulation (red) and down-regulation (blue) for each *TRIM* family gene. Top hit TRIM59 was highlighted by red arrowhead. (**B**) Quantitative analysis of TRIM59 IHC staining in 87 paired tumor/non-tumor samples. (**C**) Representative IHC staining images of adjacent normal tissues and breast cancerous tissues probed with the anti-TRIM59 antibody. A total of 154 patient samples were stained and analyzed. (**D**) Analysis of TRIM59 staining intensity in association with clinical stages of breast tumor samples (Grade I, *n* = 18; Grade I–II, *n* = 26; Grade II and higher, *n* = 110). An IHC score less than or equal to 0.75 was defined as “Low IHC ratio” or Down-regulation/No change and a value greater than 0.75 was defined as “High IHC ratio” or Up-regulation. (**E**) Kaplan–Meier survival curves for breast cancer patients based on the scores of TRIM59 IHC staining. High intensity of TRIM59 immunostaining strongly associates with poor patient survival, *P* = 0.0244. Data in **B** and **D** are presented as means ± SD; *P < 0.05, **P < 0.01, ***P < 0.001. The underlying data can be found in S1 Data. BLCA, urothelial bladder cancer; BRCA, breast invasive carcinoma; COAD, colon adenocarcinoma; ESCA, esophageal carcinoma; HNSC, heck and neck squamous cell carcinoma; IHC, immunohistochemistry; KIRC, kidney renal clear cell carcinoma; KIRP, kidney renal papillary cell carcinoma; LIHC, liver hepatocellular carcinoma; LUAD, lung adenocarcinoma; LUSC, lung squamous cell carcinoma; STAD, stomach adenocarcinoma; TCGA, the Cancer Genome Atlas; THCA, thyroid carcinoma; TRIM, tripartite motif.

The tripartite motif (TRIM) protein family of E3 ubiquitin ligases is intimately implicated in tumorigenesis by enhancing gene translocation and fusion [1,2], promoting cell cycle arrest through deregulation of tumor protein 53 (p53) and cyclin-dependent kinase (CDK) inhibitors degradation [3,4], or regulating tumor metabolic homeostasis with melanoma antigen family proteins [5]. TRIM proteins share a common domain architecture, comprising an N-terminal really interesting new gene (RING) domain, followed by one or two B-boxes (B1/B2) and a coiled-coil (CC) region. TRIM proteins often have ubiquitin (Ub) ligase activity and can selectively target Ub-modified proteins for proteasomal or autophagic degradation [6,7]. To search for potential pro-oncogenic members in the human TRIM family that are differentially expressed in cancer tissues, we systematically analyzed the expression profiles of 68 *TRIM* genes based on datasets from the Cancer Genome Atlas (TCGA) database (**Fig 1A**). Among all the *TRIM* genes, *TRIM59* was found to be the only member displaying marked up-regulation across all the 12 cancer types, a discovery that concurs with the recent findings that TRIM59 promotes the progression of prostate cancer [8], lung cancer [9], and gastric cancer [10]. While these earlier studies are primarily centered on establishing the correlations of TRIM59 with cancer hallmarks such as cell cycle progression and apoptosis, the direct targets of TRIM59 and the molecular mechanisms underpinning the pro-oncogenic role of TRIM59 in breast cancer, particularly its involvement in advanced stages of malignant transformation (cancer invasion and metastasis), remain largely unexplored.

Acquisition of invasion behavior during cancer cell metastasis involves substantial changes in cell morphology, protrusive activity, and the generation of cell polarity through controlling cytoskeletal dynamics and cell–cell adhesions [11]. Tumor cells can adopt a mesenchymal migration mode with elongated cell shapes, or display amoeboid/blebbing motility with rounded cell morphologies during metastasis. Protrusion types such as lamellipodia, driven by actin polymerization, as well as membrane blebs resulting from intracellular pressure generated by actomyosin contractions are associated with these different migration modes [12]. Rho guanosine triphosphatase (GTPase) and the Rho-associated coiled-coil kinase (ROCK) signaling have been established as central players in regulating the transition between mesenchymal and amoeboid movements. Cells with high Ras-related C3 botulinum toxin substrate 1 (Rac1) activity often display mesenchymal motility, whereas high Ras homolog family member A (RhoA) activity induces the assembly of contractile actin cortex and amoeboid (bleb) migration by promoting the phosphorylation of myosin light chain 2 (MLC2; at residues Thr18 and Ser19) [12] and the actin-membrane linkage protein, Ezrin (Thr567)/Radixin (Thr564)/Moesin (Thr558) (ERM) [13].

Among all the factors influencing the RhoA-ROCK1 signaling, the programmed cell death protein 10 (PDCD10; or cerebral cavernous malformation [CCM] 3) emerges as an upstream regulator. PDCD10 complexes with CCM1 (Krev/Rap1 Interacting Trapped 1 [KRIT1]) and CCM2 (osmosensing scaffold for mitogen-activated protein kinase kinase kinase-3 [OSM]) to control the onset of a detrimental disease named CCM. CCM is characterized by vascular lesions in the central nervous system [14]. Loss of CCM proteins, including PDCD10, has been linked to aberrant ROCK activity to cause abnormal phosphorylation of myosin light chain (MLC) and ERM. ROCK hyperactivity has been noted in CCM patients with altered expression or mutations in PDCD10 [15–20]. However, the molecular determinants that govern PDCD10 protein stability are yet to be identified. We reason that the TRIM subfamily of E3 ligases could serve as attractive candidates given their well-established roles in modulating protein degradation through multiple mechanisms, including but not limited to Ub ligation for proteasomal degradation, selective autophagy degradation pathways, or functioning as adaptors to stabilize targeted proteins by segregating them from the degradation complex [21,22]. Our study of the mechanisms by which TRIM59 controls cell plasticity through regulating PDCD10 stability and the downstream RhoA-ROCK signaling may provide novel insights into the regulatory network of cancer cell invasion and metastasis.

In this study, we identified TRIM59 as a cancer-associated E3 Ub ligase, the expression of which was strongly associated with poor clinical outcomes in breast cancer patients. TRIM59 promoted breast cancer cell growth and migration/invasion both in vitro and in vivo. Importantly, we showed that TRIM59 was essential for the transition of breast cancer cells between mesenchymal and amoeboid movements. By means of yeast two-hybrid screening, we further uncovered PDCD10 and the E3 ligase RING finger and transmembrane domain-containing protein 1 (RNFT1) [23] as direct binding partners of TRIM59. TRIM59 stabilizes PDCD10 by preventing its lysine (K) 63 ubiquitination induced by RNFT1 and the subsequent phosphotyrosine-independent ligand for the Lck SH2 domain of 62 kDa (p62)-selective autophagic degradation, thereby augmenting its suppressive effects on RhoA/ROCK1-mediated mesenchymal-amoeboid transition (MAT) in breast tumor. Our study establishes TRIM59 and PDCD10 as potential targets to develop new anticancer therapeutics.

## Results

### Up-regulation of TRIM59 in breast cancer and its correlation with poor clinical outcomes

Among the approximately 70 members in the TRIM family, TRIM59 stood out as one of the most differentially expressed genes, with its expression ubiquitously up-regulated by 1.6- to 6.3-fold in 12 analyzed cancer types compared with the adjacent normal tissues (**Figs 1A and S1A**). Particularly, in breast invasive carcinoma (BRCA), TRIM59 mRNA level was significantly increased in tumor sites (red plot) compared with its expression in normal adjacent tissues (blue plot; fold change = 4.03, *P* value = 2.9 × 10^−28^; **S1A Fig**). In parallel, we assessed the expression of TRIM59 mRNA in human and mouse tissues, as well as in a panel of human breast cancer cell lines, by using the mammary epithelial cell line MCF10A as a nonmalignant control. TRIM59 expression was relatively low in normal human and mouse mammary gland tissues when compared with cells/tissues of the immune system (spleen, thymus, T and B cells; **S1B and S1C Fig**). We further detected expression of TRIM59 in breast cancer cell lines, with the highest expression in cell lines such as MCF7 but relatively low expression in cell lines such as MDA-MB-231 (**S1D Fig**). To further verify the protein levels of TRIM59 in breast cancer patients, we performed immunohistochemistry (IHC) staining for TRIM59 on primary human breast tumors obtained from a large cohort of breast cancer patients (**S1 and S2 Tables**). Among the 154 patients, 87 biopsy specimens contained both tumors and matched normal adjacent tissues, whereas the other 67 had only tumor tissues. Among the 87 matched samples, we detected markedly higher intensities of TRIM59 immunostaining in breast tumors than in tumor-adjacent normal tissues (**Fig 1B and 1C and S1 and S2 Tables**). Notably, TRIM59 protein levels were positively correlated with the pathologic grades of breast cancer (Grade I, *n* = 18; Grade I–II, *n* = 26; Grade II and higher, *n* = 110) and were slightly higher in estrogen receptor (ER)-negative breast cancer (**Figs 1D and S1E–S1G and S1 and S2 Tables**). Most clinically relevant, elevated TRIM59 expression was significantly associated with shortened breast cancer patient survival (*P* = 0.0244) (**Fig 1E**). To confirm that TRIM59 is an independent factor linked to clinical outcomes, we performed multivariate overall survival analysis by using a Cox proportional hazard model based on available clinical information, including patient cancer stages and pathological grades. Our statistical analysis confirmed TRIM59 expression as an independent prognostic factor (hazard ratio = 2.99; 95% CI: 1.10–8.12, *P* = 0.031, **S1H Fig**). In addition, even among high-grade (Grade II and higher) patients, higher TRIM59 expression was significantly correlated with a shortened survival rate (log-rank test *P* value = 0.017, **S1I Fig**). Together, our results establish a positive correlation of augmented TRIM59 expression with breast cancer progression and survival.

### TRIM59 promotes breast tumor growth both in vitro and in vivo

**Fig 2:**
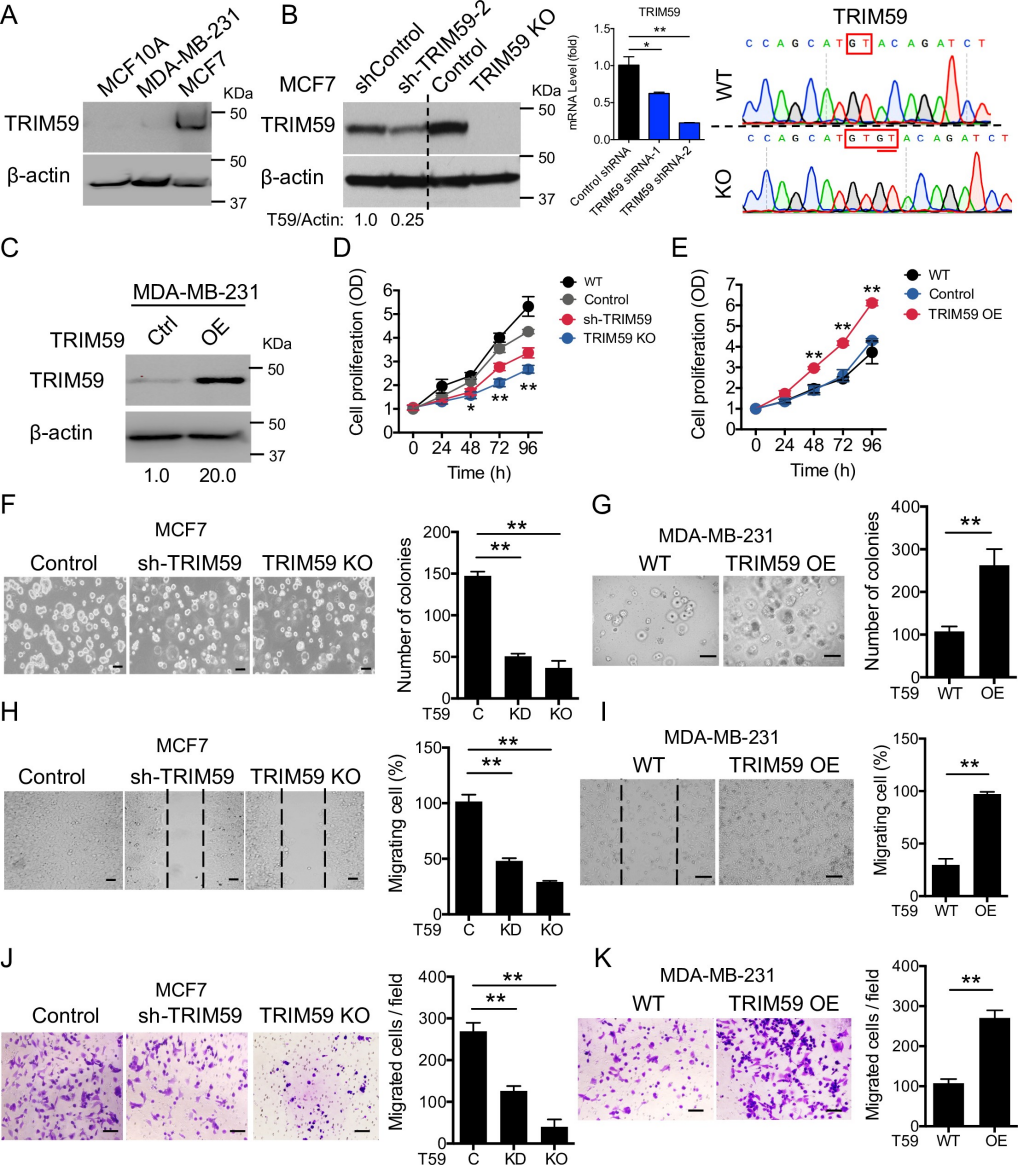
TRIM59 promotes breast cancer cell proliferation, growth, migration, and invasion in vitro. (**A**) IB analysis of TRIM59 expression in the noncancerous mammary epithelial cell line (MCF10A) and breast cancer cell lines (MCF7 and MDA-MB-231). (**B** and **C**) IB analysis of TRIM59 expression in shRNA or CRISPR/Cas9 lentivirus-transduced control, *TRIM59* KD (sh*TRIM59*-2, KD efficiency confirmed by qPCR; **B**, middle) and *TRIM59* KO MCF7 (**B**, left), or TRIM59-overexpressing (OE) MDA-MB-231 stable cells (**C**). sgRNA targeting TRIM59 was confirmed by Sanger sequencing (**B**, right). A dinucleotide GT insertion (underlined) caused an early abortion of TRIM59 translation, as further validated by immunoblotting (**B**, lane 4). (**D** and **E**) Graphic representation of 96-hour MTS proliferation assays of WT (non-transduced), control (transduced with non-targeting shRNA/sgRNA), sh*TRIM59* and *TRIM59* KO MCF7 cells (**D**), and WT or TRIM59 OE MDA-MB-231 cells (**E**). (**F** and **G**) Colony formation assay in 3D Matrigel culture was performed to assess the rate of colony formation of control, sh*TRIM59*, and *TRIM59* KO MCF7 cells at day 8 (**F**) or WT and TRIM59 OE MDA-MB-231 cells at day 4 (**G**). Bar graphs show the number of colony-forming units (*n* = 8). Scale bars: 100 μm. (**H** and **I**) Scratched wound healing assays were performed to assess the rates of migration for control, sh*TRIM59*, and *TRIM59* KO MCF7 cells at 72 hours (**H**) or WT and TRIM59 OE MDA-MB-231 cells at 36 hours (**I**). Bar graphs show the percentage of migration rate (*n* = 8). Scale bars: 100 μm. (**J** and **K**) Transwell invasion assay was performed in Matrigel-coated 8-μm pore size transwell plates to assess the invasion ability of control, sh*TRIM59*, and *TRIM59* KO MCF7 cells (**J**) or WT and TRIM59 OE MDA-MB-231 cells (**K**). Bar graphs show the numbers of invaded cells (*n* = 8). Scale bars: 100 μm. Data in **B**, **D-K** are presented as means ± SD; *P < 0.05, **P < 0.01 versus controls. The underlying data can be found in S1 Data. CRISPR/Cas9, clustered regularly interspaced short palindromic repeats (CRISPR)-associated protein-9 nuclease; IB, immunoblot; KD, knockdown; KO, knockout; MTS, 3-(4,5-dimethylthiazol-2-yl)-5-(3-carboxymethoxyphenyl)-2-(4-sulfophenyl)-2H-tetrazolium; OD, optical density; OE, overexpressing; qPCR, quantitative polymerase chain reaction; sgRNA, single guide RNA; shRNA, short hairpin RNA; TRIM59, tripartite motif 59; WT, wild-type.

To examine the biological function of TRIM59 in breast cancer, we employed a short hairpin RNA (shRNA)-based knockdown (KD by 75%, **Fig 2B**) or clustered regularly interspaced short palindromic repeats (CRISPR)-associated protein-9 nuclease (CRISPR/Cas9)-based knockout (KO, guanine- thymine [GT] insertion, **Fig 2B**) strategy in TRIM59-high breast cancer cell line MCF7 (**Figs 2A and S2A and S2B**). In parallel, we stably overexpressed TRIM59 by about 20-fold (**Fig 2C**) in MDA-MB-231, a breast cancer cell line with relatively low TRIM59 expression (**Figs 2A and S2A and S2B**). Using a cell proliferation colorimetric assay, we found that *TRIM59* KD or KO in MCF7 pronouncedly suppressed cell proliferation (with both nontransfected wild-type [WT]) and control shRNA (shControl)-transfected cells as controls; **Fig 2D**). By contrast, cell proliferation was significantly enhanced by overexpressing (OE) TRIM59 in MDA-MB-231 cells (**Fig 2E**). We next examined the anchorage-independent growth of *TRIM59* KD/KO MCF7 cells and TRIM59 OE MDA-MB-231 cells by using a Matrigel-based 3D culture system. *TRIM59* depletion dramatically reduced the frequency of colony formation of MCF7 cells from 14.7 ± 0.5% (control) to 5.1 ± 0.3% (sh*TRIM59*) and 3.7 ± 0.9% (*TRIM59* KO) (**Fig 2F**). Conversely, TRIM59 overexpression promoted the colony formation of MDA-MB-231 cells from 10.8 ± 0.1% to 26.2 ± 0.4% (**Fig 2G**). Furthermore, TRIM59 inactivation dramatically impaired MCF7 cell migration by 50%–60% (**Fig 2H**), whilst TRIM59 overexpression increased cell migration of MDA-MB-231 cells by 60% (**Fig 2I**). We further assessed the impact of TRIM59 inactivation or overexpression on cell migration and invasion using an independent assay based on Matrigel-coated transwell chambers. Again, down-regulation or loss of *TRIM59* impeded MCF7 cell invasion (**Fig 2J**), whereas TRIM59 overexpression increased the numbers of invaded MDA-MB-231 cells (**Fig 2K**). Our findings establish that TRIM59 promotes the proliferation, migration, and invasion of breast cancer cells.

**Fig 3:**
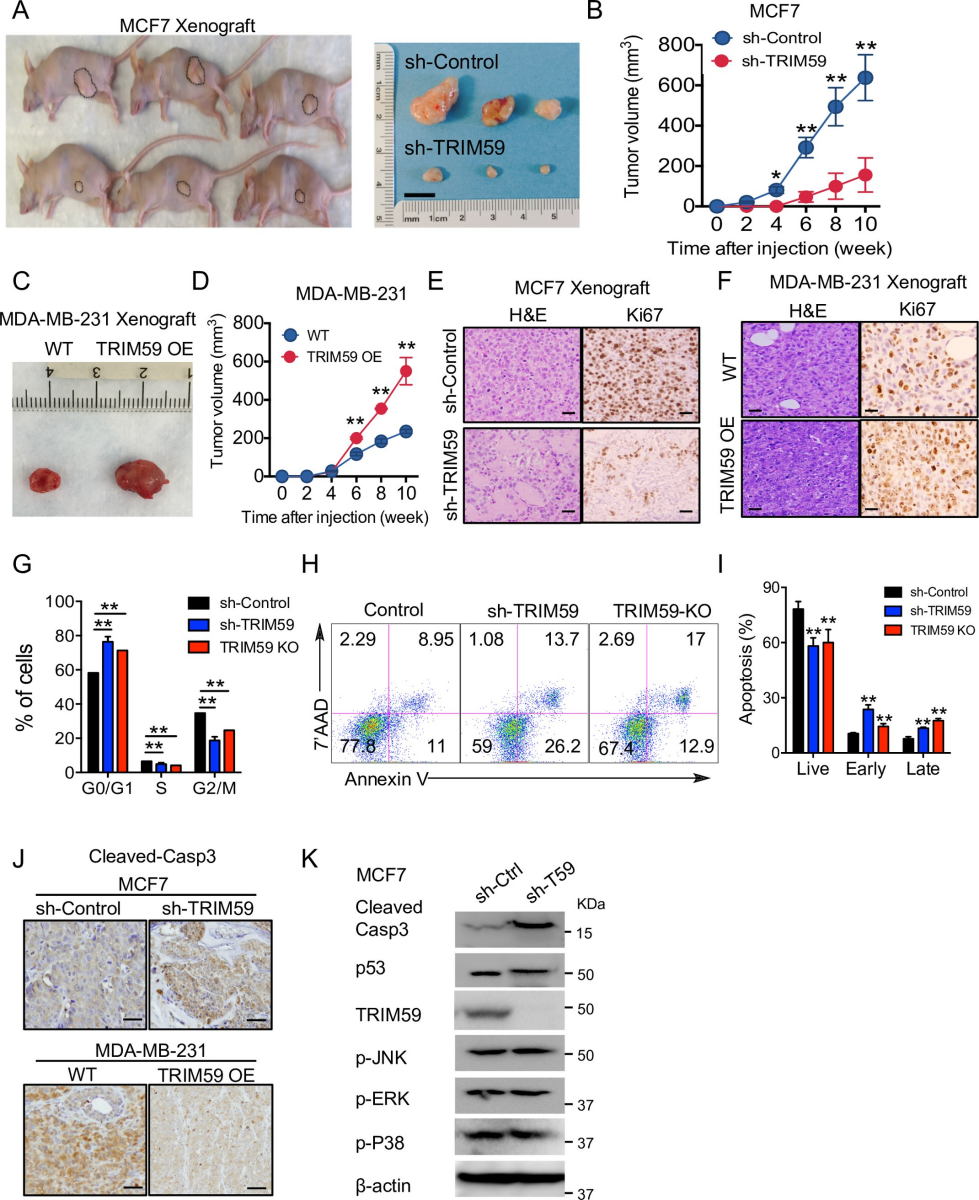
TRIM59 promotes the tumor growth and survival of breast cancer cells in vivo. (**A** and **B**) Representative tumor images (**A**) and tumor volume at indicated time points (**B**) in nude mice that were orthotopically injected into mammary fat-pad with control (non-targeting shRNA) or shTRIM59 MCF7 cells (1 × 10^7^ cells/mouse). *n* = 9 mice per group. (**C** and **D**) Representative tumor images (**C**) and tumor size quantification at indicated time points (**D**) in NSG mice that were orthotopically injected into mammary fat-pad with control and TRIM59 OE MDA-MB-231 cells (5 × 10^6^ cells/mouse). *n* = 5 mice per group. (**E** and **F**) Representative image of shControl or sh*TRIM59* MCF7 xenografts (**E**) and control or TRIM59 OE MDA-MB-231 (**F**) xenograft tumors stained with HE and Ki-67. Scale bars: 20 μm. (**G**) Cell cycle analysis on Control, sh*TRIM59*, and *TRIM59* KO MCF7 cells by flow cytometry. The population of cells at each stage was shown in the bar graph. *n* = 6. **P < 0.01 versus control. (**H** and **I**) Apoptotic assessment of shControl, sh*TRIM59*, and *TRIM59* KO MCF7 cells. Apoptosis was assayed with Annexin V/7-AAD staining and measured by flow cytometry (**H**). Statistical analysis of cell populations at early or late stages of apoptosis (**I**). Live, 7’AAD^−^AnnexinV^−^; Early, 7’AAD^−^AnnexinV^+^; Late, 7’AAD^+^AnnexinV^+^. *n* = 6. (**J**) Representative images of cleaved-caspase3 expression in paraffin sections from xenograft tumors made of shControl or sh*TRIM59* MCF7 cells (top) and WT or TRIM59 OE MDA-MB-231 cells (bottom). *n* = 6. Scale bars: 10 μm. (**K**) IB analysis of cell cycle/apoptosis regulators (p53 and MAP kinase pathway members) expression in shControl or sh*TRIM59* MCF7 cells. Data in **B**, **D**, **G**, and **I** are presented as means ± SD. *P < 0.05, **P < 0.01 versus controls. The underlying data can be found in S1 Data. Casp3, caspase3; HE, hematoxylin–eosin; Ki-67, marker of proliferation; KO, knockout; MAP, mitogen-activated protein; NSG, NOD scid gamma; OE, overexpressed; p-ERK, phosphorylation of extracellular signal–regulated kinase (ERK); p-JNK, phosphorylation of c-Jun N-terminal kinase (JNK); p-P38, phosphorylation of P38 MAP kinase; p53, tumor protein 53; shRNA, short hairpin RNA; TRIM59, tripartite motif 59; WT, wild-type.

In order to investigate the functional consequence of TRIM59 inactivation or overexpression in vivo, we orthotopically injected MCF7 cells and MDA-MB-231 cells stably expressing sh*TRIM59* or TRIM59-internal ribosome entry site-green fluorescent protein (TRIM59-IRES-GFP), respectively, with Matrigel (1:1) into the NOD scid gamma (NSG) mouse mammary fat-pad and monitored tumor growth up to 10 weeks. The tumor weight and volume were substantially reduced in mice bearing *TRIM59* KD MCF7 cells when compared with control mice (**Fig 3A and 3B**). By comparison, mammary xenograft tumor growth was greatly accelerated in mice bearing TRIM59 OE MDA-MB-231 cells, with a notable increase in tumor sizes (**Fig 3C and 3D**). IHC staining of the marker of proliferation (Ki-67), revealed fewer proliferative cells in *TRIM59* KD xenograft tumors (**Fig 3E**) but more prominent proliferative activity in TRIM59 OE MDA-MB-231 xenografts, when compared with the control samples (**Fig 3F**). Collectively, results from our in vivo studies further reinforced the conclusion that TRIM59 plays a pro-oncogenic role in breast cancer by promoting tumor cell growth and migration.

### TRIM59 regulates breast cancer cell apoptosis and cell cycle independent of p53

To understand the molecular mechanisms by which TRIM59 promotes breast tumor growth, we performed quantitative functional proteomics studies on MCF7 cells (*TRIM59* KD or KO versus control) by using a high-throughput reverse phase protein array (RPPA) [24]. Our unbiased comparative analysis revealed the differential expression/phosphorylation of at least nine key cancer-associated signaling proteins (**S2C Fig**), which might be directly or indirectly affected upon alteration of TRIM59. For instance, we detected the up-regulation of G1/S phase negative regulators, CDK inhibitors p21 and p27, and the down-regulation of a positive regulator kinase CDK1. These changes to some extent explained our observation that MCF7 cells manifested an apparent accumulation at the G0/G1 phase following *TRIM59* depletion (**Fig 3G**). In TRIM59-depleted MCF7 cells, we also observed down-regulation of pro-oncogenic factors, such as avian myelocytomatosis virus oncogene cellular homolog (c-Myc), type I insulin-like growth factor receptor (IGF1R), and vascular endothelial growth factor receptor 2 (VEGFR2), along with enhanced expression of E-cadherin (encoded by cadherin 1 [Cdh1]) and inositol polyphosphate-4-phosphatase type II b (INPP4b), a recently discovered tumor suppressor [25–27] (**S2C Fig**).

We next assessed the apoptotic properties of breast cancer cells by using 7’AAD and Annexin V double staining. *TRIM59*-depleted MCF7 cells exhibited higher frequencies of early-stage (indicated by the Annexin V+ and 7’AAD− population) and late-stage apoptosis (Annexin V+7’AAD+ staining) (**Fig 3H and 3I**). Consistently, xenograft tumors isolated from mice injected with *TRIM59* KD MCF7 cells showed substantially higher staining of cleaved-caspase3. By contrast, TRIM59-OE MDA-MB-231 cells displayed reduced staining of cleaved-caspase3, an indicator of active apoptosis (**Fig 3J**). Because the rat sarcoma (Ras)-mitogen-activated protein kinase (MAPK) signaling [28] and p53 signaling [29] are most commonly implicated in the regulation of cell cycle and apoptosis, we further asked whether these signaling components were altered in *TRIM59*-depleted MCF7 cells. We set out to test this by determining the protein levels of p53 and cleaved caspase 3, as well as the phosphorylation status of p38, ERK1/2, and c-Jun N-terminal kinase (JNK) in *TRIM59* KD MCF7 cells, with immunoblotting. We did not observe significant changes in expression or phosphorylation between *TRIM59* KO and WT MCF7 cells (**Fig 3K**). In aggregate, our findings suggest that TRIM59-mediated regulation of breast cancer cell growth is very likely independent of the MAPK and p53 signaling pathways.

### TRIM59 facilitates breast cancer cell metastasis

**Fig 4:**
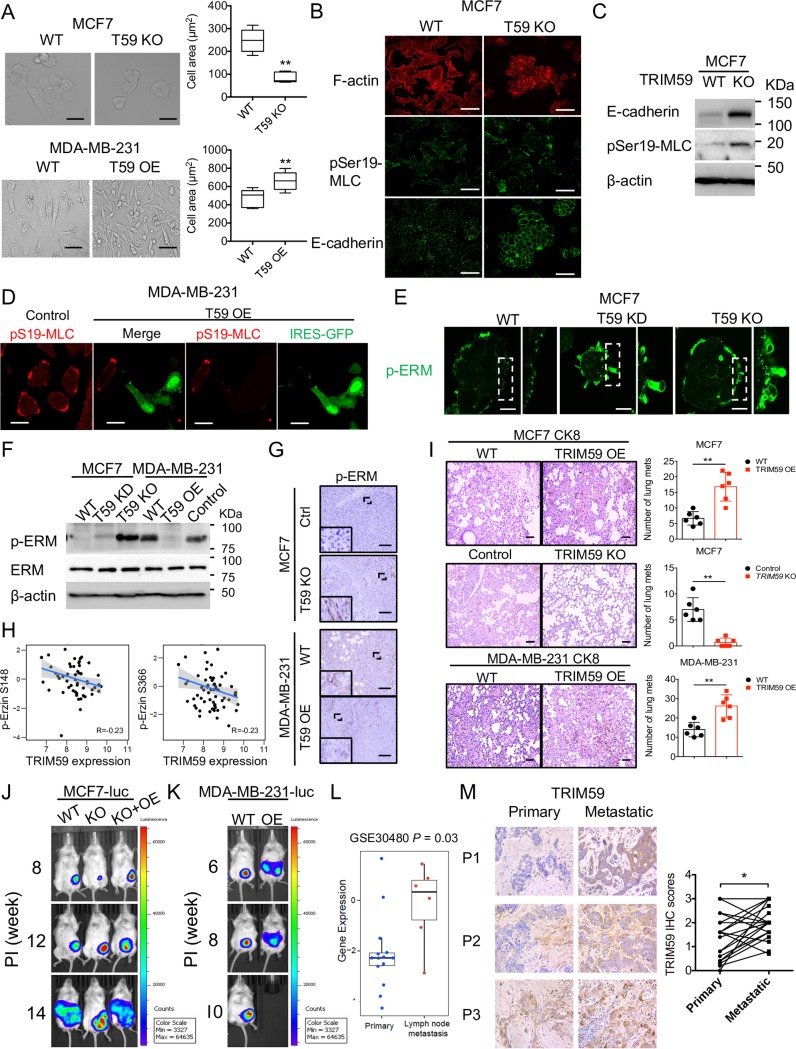
TRIM59 regulates breast cancer cell actomyosin contractility and metastasis. (**A**) Representative images of cell morphology changes upon TRIM59 deletion (T59 KO) in MCF7 cells (upper, left) or overexpression (T59 OE) in MDA-MB-231 cells (bottom, left). Scale bars: 20 μm. Quantification of the cell area of MCF7 (upper, right) and MDA-MB-231 cells (bottom, right). *n* = 6. (**B**) Representative confocal images of MCF7 cells depleted of TRIM59 and stained for F-actin, pSer19-MLC, and E-cadherin. Scale bars: 20 μm. (**C**) IB analysis of E-cadherin, pSer19-MLC expression in WT and TRIM59-KO MCF7 cells. (**D**) Representative confocal images of TRIM59 overexpressing (T59 OE) MDA-MB-231 cells with IRES-GFP stained for pSer19-MLC (red). Scale bars: 10 μm. (**E**) Representative confocal images of p-ERM expression (green) in WT and *TRIM59-*deleted MCF7 cells. Scale bars: 10 μm. (**F**) IB analysis of p-ERM and total ERM levels in WT, TRIM59 KD, or TRIM59 KO MCF7 cells (lanes 1–3) and WT or TRIM59 OE MDA-MB-231 cells (lanes 4–6). (**G**) Representative IHC staining images for p-ERM in tissue sections from xenograft tumors of TRIM59 KO MCF7 cells or TRIM59 OE MDA-MB-231 cells compared with control cells. Scale bars: 200 μm. (**H**) Spearman correlation between TRIM59 expression and ezrin phosphorylation (p-S148, p-S366) levels. (**I**) Representative images showing HE and CK8 staining of lung micro-metastases of xenograft tumors in the indicated groups (WT versus TRIM59 OE or TRIM59 KO in MCF7 or MDA-MB-231 cells). Scale bars: 100 μm (HE); 20 μm (CK8). Red arrow: pulmonary metastatic foci. Statistical analysis of the number of lung metastases was shown in the right panel. *n* = 6. (**J** and **K**) Representative luminescent images of the primary and metastases acquired with an IVIS imaging system. Luminescence imaging in NSG mice was followed after inoculation with breast cancer cells into the mammary fat-pad at the indicated time points (*n* = 3). Note, 10-week-old NSG mice inoculated with TRIM59 OE MDA-MB-231-luc died without bioluminescent imaging. (**L**) Gene expression analysis of *TRIM59* in primary and lymph node metastases (GSE30480 dataset). (**M**) Representative TRIM59 IHC staining images in paired primary breast cancer and metastatic lymph nodes from breast cancer patients (*n* = 19). Statistical analysis of paired patient samples (primary versus metastatic tissues in the same patient) were shown on the right. Data in **A**, **I**, **L**, and **M** are presented as means ± SD. *P < 0.05, **P < 0.01 versus controls. The underlying data can be found in S1 Data. CK8, cytokeratin 8; ERM, Ezrin (Thr567)/Radixin (Thr564)/Moesin (Thr558); HE, hematoxylin–eosin; IB, immunoblot; IHC, immunohistochemistry; IRES, internal ribosome entry site; GFP, green fluorescent protein; IVIS, in vivo imaging system; KD, knockdown; KO, knockout; Min, minimum; Max, maximum; NSG, NOD scid gamma; OE, overexpressed; p-ERM, phosphorylation of ERM; pSer19-MLC, phosphorylation of MLC at serine 19; TRIM59, tripartite motif 59; WT, wild-type.

Upon *TRIM59* depletion or overexpression, we noticed prominent morphological changes in breast cancer cells. *TRIM59* depletion in MCF7 cells caused a pronounced reduction of cell area with increased cell–cell interactions. By contrast, overexpression of TRIM59 changed the overall shape of MDA-MB-231 cells into a more elongated, mesenchymal phenotype, with longer membrane protrusions and reduced cell–cell adhesion (**Fig 4A**). Cell shape is mainly controlled by the organization of cytoskeleton and actin/myosin (actomyosin)-mediated cell contractility [30], which can be monitored by assessing the phosphorylation of MLC (p-MLC) and ERM (p-ERM; Ezrin-Radixin-Moesin). Cytoskeletal F-actin staining with fluorescent phalloidin confirmed the morphological change of MCF7 cells upon *TRIM59* depletion. In addition, depletion of *TRIM59* in MCF7 cells changed the organization of p-MLC to generate an accumulated arc of p-MLC in the cell cortex, accompanied by increased staining of E-cadherin, which mediates cell–cell adhesion (**Fig 4B**). In line with the immunostaining results at single cell levels, immunoblot (IB) analysis revealed an increase in the total amounts of phosphorylated MLC and E-cadherin (**Fig 4C**). Conversely, overexpression of TRIM59 (with IRES-GFP) in MDA-MB-231 cells led to a marked loss of p-MLC staining compared to non-overexpressing (GFP-negative) cells in the same imaging field (**Fig 4D**). We next examined the effect of altered TRIM59 expression on another indicator of cell contractility by assessing the phosphorylation status of ERM with immunostaining and western blotting. Upon *TRIM59* deletion, we observed enhanced blebbing (**Fig 4E**), as well as increased p-ERM levels, but no significant changes in the total ERM levels in both MCF7 cells (**Fig 4F**) and xenograft tumors (**Fig 4G**). In contrast, TRIM59 overexpression in MDA-MB-231 cells suppressed ERM phosphorylation (**Fig 4F and 4G**). In human samples, Spearman correlation analysis based on Clinical Proteomic Tumor Analysis Consortium (CPTAC) [31] also revealed a negative correlation between TRIM59 expression and phosphorylation of ezrin (**Fig 4H**, p-S148 and p-S366, *R* = −0.23). These data suggest that TRIM59 modulates phosphorylation of MLC and ERM in breast cancer cells. In the xenograft models, overexpression of TRIM59 in MCF7 or MDA-MB-231 cells promoted pulmonary metastasis of breast cancer cells, whereas *TRIM59* deficiency suppressed cancer cell metastasis, as reflected by cytokeratin 8 (CK8) staining of metastasized cancer cells in the lung (**Fig 4I**). We next performed in vivo imaging analysis to monitor the effect of TRIM59 on tumor metastasis in NSG mice injected with MCF7-luc or MDA-MB-231-luc cells (luciferase-expressing stable cells). *TRIM59* deficiency suppressed primary tumor growth at early stages (around 8 weeks postinjection). This phenotype could be reversed through the reconstitution of TRIM59 OE (KO+OE group; **Fig 4J**). Notably, *TRIM59*-deficient MCF7-luc cells acquired a growth advantage later (around 12 weeks postinjection, KO tumor size was comparable to control or KO+OE group), but the metastatic ability was greatly reduced (**Fig 4J**, middle). Re-expression of TRIM59 restored the metastatic capacity of MCF7-luc cells to a level comparable to the WT group (**Fig 4J**, right). Similarly, overexpression of TRIM59 in MDA-MB-231-luc cells promoted cancer cell metastasis at approximately 6 weeks postinjection and greatly shortened survival, because the mice died at 10 weeks (**Fig 4K**). We further analyzed the expression of TRIM59 in human breast cancer patient lymph node metastases versus primary tumor with both RNA sequencing dataset analysis (GSE30480) and IHC staining on patient tissue array samples (paired primary versus metastatic tissues from the same patient). We found a significantly higher expression of TRIM59 in metastatic breast cancer at both mRNA and protein levels (**Fig 4L and 4M**). Thus, our data demonstrate a positive correlation between TRIM59 expression and breast cancer metastasis.

Because β-catenin is also a component for the cancer-promoting Wnt signaling that is intimately involved in tumor metastasis [32–35], we further examined the expression of β-catenin and Wnt target gene AXIN2 [36]. Imaging of β-catenin staining in MCF7 and MDA-MB-231 xenografts revealed that TRIM59 was positively correlated with the expression of β-catenin, while AXIN2 transcription was suppressed by *TRIM59* deletion (**S3A and S3B Fig**). These findings indicate that TRIM59 might also promote β-catenin expression and the activation of the Wnt pathway.

Together, our data indicate that TRIM59 promotes breast cancer cell metastasis by (1) inhibiting the phosphorylation of MLC and ERM required for actomyosin contractility and amoeboid migration, (2) reducing the expression of cell adhesion molecules such as E-cadherin, and (3) increasing the cellular level of β-catenin and promoting metastasis-associated Wnt signaling.

### TRIM59 binds to PDCD10 and blocks PDCD10 autophagic degradation

**Fig 5:**
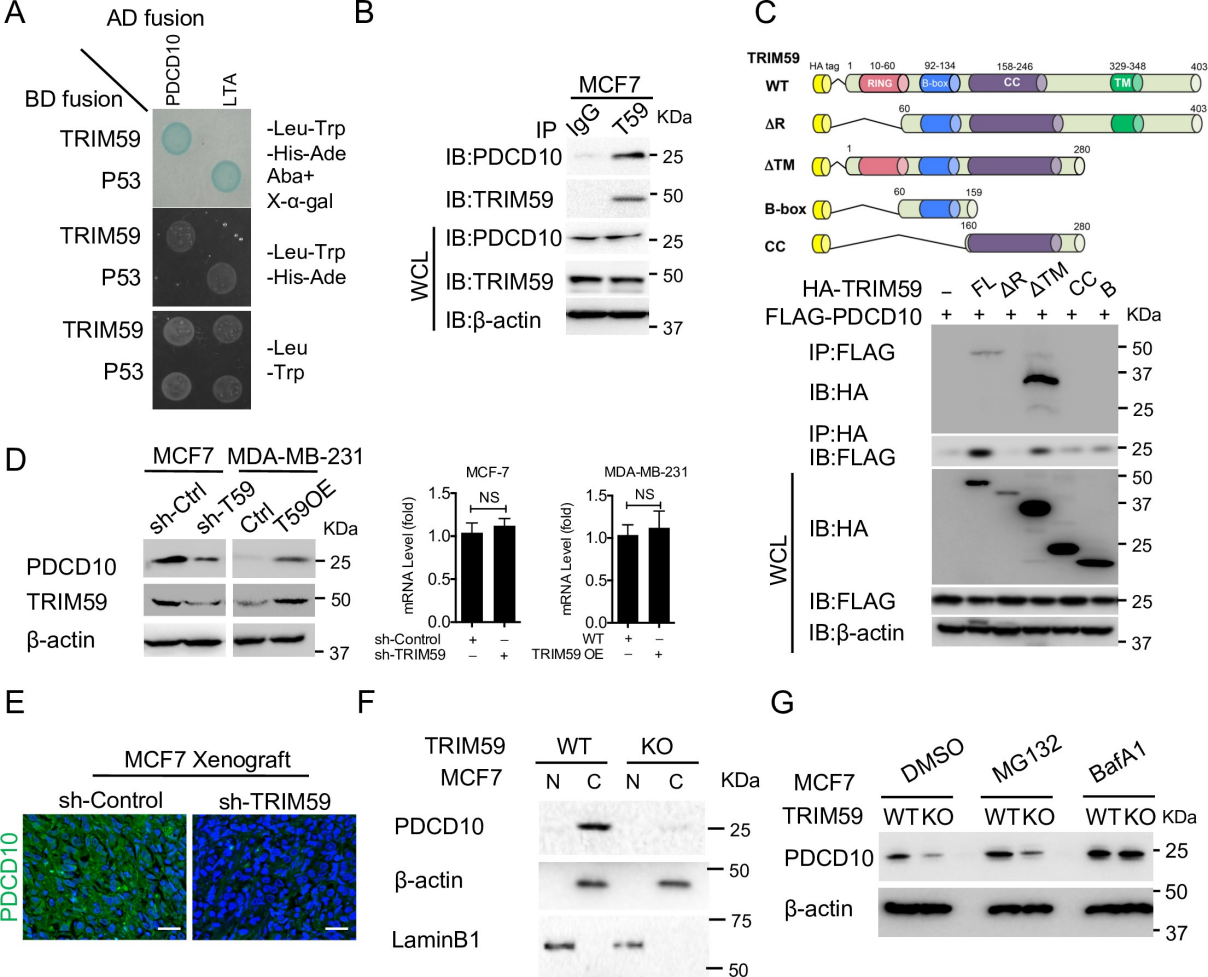
TRIM59 interacts with PDCD10 and blocks the autophagic degradation of PDCD10. (**A**) Yeast two-hybrid screening to confirm the interaction between TRIM59-BD and PDCD10-AD. Transformed and mated cells were grown on–Leu–Trp medium (*bottom*). Interaction of TRIM59 with PDCD10 was verified on high-stringency plates (–Leu–Trp–His–Ade) and with a β-galactosidase filter assay and Aureobasidin A (AbA) (*top*). Interaction of p53 and SV40 large T-antigen (LTA) was used as positive control. (**B**) Co-IP and IB analysis of extracts of MCF7 cells with the indicated antibodies. IP with IgG was added as a negative control. (**C**) Co-IP and IB analysis of extracts of HEK293T cells transfected with FLAG-PDCD10 along with HA-tagged TRIM59 variants (full-length WT or deletions [ΔR, ΔTM, B, and CC] as shown on the top cartoon). (**D**) IB and qPCR analysis of PDCD10 protein expression (left) and mRNA levels (right) in shControl and sh*TRIM59* MCF7 cells (**D**, lane 1 versus 2) or in control and TRIM59 OE MDA-MB-231 cells (**D**, lane 3 versus 4). Data are presented as means ± SD. NS versus controls. The underlying data can be found in S1 Data. (**E**) Representative immunofluorescent staining of PDCD10 (green) in sections from xenograft of shControl or sh*TRIM59* MCF7 cells. DAPI, DNA-intercalating dye, indicates the nucleus (blue). Scale bars: 100 μm. *n* = 4. (**F**) IB analysis of PDCD10 expression in MCF7 WT or TRIM59 KO cell fractions. β-actin and LaminB1 were used as markers for the cytosolic and nuclear fractions, respectively. (**G**) IB analysis of PDCD10 expression in WT or TRIM59 KO MCF7 cells treated with DMSO or 10 μM MG132 (proteasome inhibitor) or 200 nM BafA1 (autophagy inhibitor). See **S4C Fig** for similar experiments using TRIM59 KD cells. ΔR, TRIM59 without RING domain; ΔTM, TRIM59 without the predicted transmembrane domain; AbA, Aureobasidin A; AD, transcription activation domain;–Ade, adenine dropout; B, B-box-type zinc finger domain; BafA1, bafilomycin A1; BD, DNA-binding domain; C, cytosolic fraction; CC, coiled-coil domain; co-IP, co-immunoprecipitation; HA, hemagglutinin;–His, histidine dropout; IB, immunoblot; IgG, immunoglobulin G; IP, immunoprecipitation; KD, knockdown; KO, knockout;–Leu, Leucine dropout; LTA, large T-antigen; N, nuclear fraction; NS, not significant; OE, overexpressing; PDCD10, programmed cell death protein 10; p53, tumor protein 53; qPCR, quantitative polymerase chain reaction; SV40, polyomavirus simian virus 40; TM, transmembrane domain; TRIM59, tripartite motif 59;–Trp, tryptophan dropout; WT, wild-type.

To gain insight into the direct target(s) of TRIM59 in the regulation of breast cancer cell survival and metastasis, we set out to perform an unbiased yeast two-hybrid screening assay (p53 and Large T-antigen as positive control) by using TRIM59 as prey and normalized universal human cDNA library as bait. PDCD10 stood out as a strong candidate that was repeatedly detected in the 40+ positive clones after stringent selections (**Fig 5A and S3 Table**). PDCD10 has been recently shown to suppress Rho/ROCK-mediated actomyosin contractility [15], thus prompting us to hypothesize that PDCD10 might serve as the key player to mediate the TRIM59-related conversion of cell migration modes.

To further biochemically validate the endogenous TRIM59-PDCD10 interaction, we performed a co-immunoprecipitation (co-IP) assay in MCF7 cells. After pulling down with an anti-TRIM59 antibody, we detected the co-enrichment of PDCD10 by blotting with an anti-PDCD10 antibody (**Fig 5B,** immunoglobulin G [IgG] as a negative control). To further narrow down the key domains within TRIM59 that mediate the interaction with PDCD10, we generated a set of truncated or deleted variants of TRIM59 (**Fig 5C**), including the full-length TRIM59 (FL), TRIM59 without RING (R) domain (ΔR), TRIM59 without the predicted transmembrane domain (ΔTM), coiled-coil domain (CC), and B-box only domain (B). We found that PDCD10 interacted with FL and ΔTM, and that deletions of the RING domain (in ΔR, CC, and B constructs) compromised the PDCD10-TRIM59 interaction. These findings suggest that the RING domain within TRIM59 is essential for its physical interaction with PDCD10 (**Fig 5C**).

Because the RING domains of E3 ligases are often involved in mediating protein ubiquitination and degradation [37,38], we reasoned that TRIM59 might modulate the protein stability of PDCD10. After knocking down *TRIM59* using two different shRNAs, we unexpectedly found that the expression of endogenous PDCD10 was greatly reduced in MCF7 cells (**Fig 5D**, left panel lane 2, **S4A and S4B Fig**). The down-regulation of PDCD10 expression at protein levels was further validated by immunofluorescent staining in *TRIM59* KO MCF7 xenograft tumors (**Fig 5E**). Conversely, accumulation of PDCD10 was observed upon overexpression of TRIM59 in MDA-MB-231 cells (**Fig 5D**, left panel lane 4). Under all these conditions, the mRNA levels of *PDCD10* remained largely unaltered (**Fig 5D**, right panel), thus ruling out a transcriptional regulatory mechanism. Furthermore, to rule out the possibility that TRIM59 might promote the nuclear translocation of PDCD10 to prevent its degradation in the cytoplasm, we separated the nucleus and cytoplasm fractions of WT or *TRIM59* KO MCF7 cells. We found that PDCD10 is a cytoplasmic protein and its protein level is greatly reduced upon *TRIM59* deletion (**Fig 5F**). Thus, TRIM59 is essential for the maintenance of steady-state levels of PDCD10 protein.

To identify the protein degradation pathways that might mediate PDCD10 stability upon *TRIM59* depletion or overexpression, we first examined the protein stability of PDCD10 in *TRIM59* KD (**S4C Fig**) or *TRIM59* KO (**Fig 5G**) MCF7 cells in the presence of a proteasome inhibitor, MG132, and autophagy inhibitor bafilomycin A1 (BafA1) or 3-methyladenine (3-MA), as well as DMSO as control. We found that inhibition of autophagy with BafA1 or 3-MA restored PDCD10 protein levels, even upon *TRIM59* depletion, but not with the proteasome inhibitor MG132 (**Figs 5G and S4C and S4E**). In parallel, we overexpressed TRIM59 in TRIM59-low MB-MDA-231 cells, with and without treatment of BafA1, and monitored PDCD10 protein levels by immunoblotting. We found that ectopic expression of TRIM59, similar to BafA1 treatment, caused a significant up-regulation of PDCD10 protein levels (**S4D Fig,** lanes 2 and 3 versus 1). In addition, the TRIM59 OE group in the presence of BafA1 did not show further up-regulation of PDCD10 (**S4D Fig,** lane 4 versus 3). Notably, depletion of *TRIM59* did not seem to affect the basal autophagosome formation, as reflected by the similar puncta areas or numbers of autophagosomal marker GFP-microtubule-associated protein 1A/1B-light chain 3 (LC3) (GFP-LC3) [39] per cell in WT and the *TRIM59* KO human embryonic kidney 293 cell line that expresses a mutant version of SV40 large T antigen (HEK293T) cells (**S4F Fig**). Collectively, our results establish that TRIM59 could prevent PDCD10 from autophagic degradation.

### TRIM59 suppresses cell contractility through PDCD10-mediated inhibition of RhoA-ROCK1 signaling

**Fig 6:**
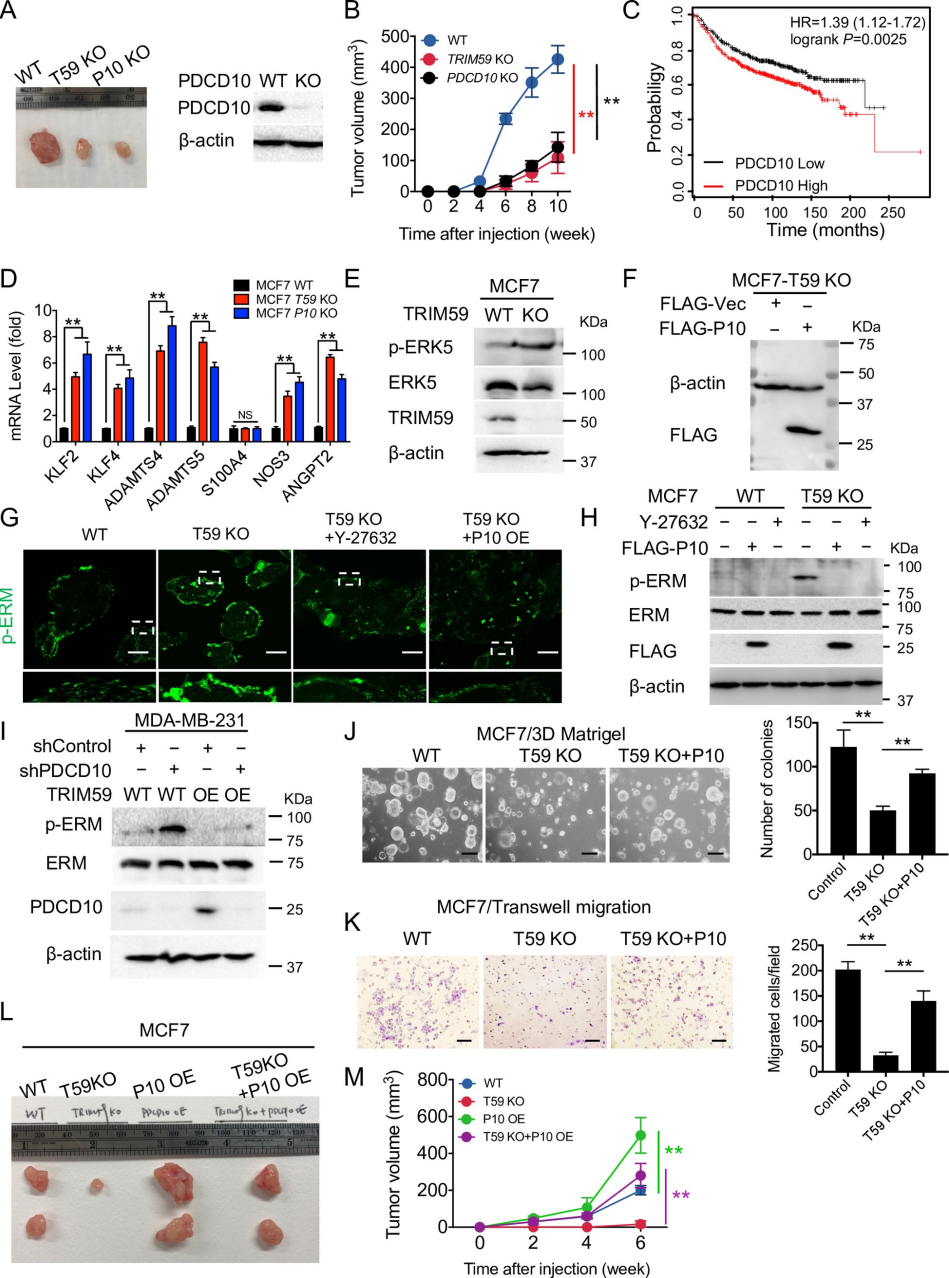
TRIM59 suppresses cell actomyosin contractility through PDCD10-mediated inhibition of RhoA-ROCK1 signaling. (**A** and **B**) Representative xenograft images of MCF7 cells depleted with TRIM59 or PDCD10 compared with WT control (**A**) and tumor volume at indicated time points (**B**) in NSG mice; 5 × 10^6^ cells/mouse. *n* = 4 mice per group. IB analysis of PDCD10 expression in WT and KO MCF7 cells was shown (**A**, right). (**C**) Kaplan–Meier survival curves were analyzed based on the expression of PDCD10 (Affymetrix ID: 210907_s_at), according to an online survival analysis tool using breast cancer microarray data (see Materials and methods for more details). *n* > 100. *P* = 0.0025. (**D**) Quantitative PCR to evaluate the expression of PDCD10 downstream genes in WT, *TRIM59* KO, and *PDCD10* KO MCF7 cells. *n* = 4. (**E**) IB analysis of phosphorylation of ERK5, total ERK5, and TRIM59 expression in WT and TRIM59 KO MCF7 cells. (**F**) IB analysis of FLAG-PDCD10 expression in MCF7 TRIM59 KO cells transduced with a lentiviral expression construct expressing FLAG-PDCD10. (**G** and **H**) Representative immunofluorescent staining (**G**, *n* = 3) or IB analysis (**H**) of p-ERM and total ERM in WT or TRIM59 KO MCF7 cells treated with RhoA-ROCK1 signaling inhibitor Y-27632 or ectopic expression of PDCD10 (P10 OE). Scale bars: 20 μm. (**I**) IB analysis of p-ERM and total ERM in WT or TRIM59 overexpressing MDA-MB-231 cells transduced with control shRNA or *PDCD10* shRNA (sh*PDCD10*). (**J** and **K**) Colony formation assay in 3D Matrigel culture (**J**) and Transwell invasion assay (**K**) were performed to assess the colony formation and invasion of WT, TRIM59 KO MCF7 cells with or without ectopic expression of FLAG-PDCD10. Bar graphs showed the number of colony-forming units (**J**) and the number of invaded cells (**K**) (*n* = 5). Scale bars: 10 μm (**J**); 100 μm (**K**). (**L** and **M**) Representative xenograft images of WT, *TRIM59-*depleted (T59 KO), PDCD10 overexpressing (P10 OE), and T59 KO reconstituted with P10 OE (T59 KO+P10 OE) MCF7 cells (**L**) and tumor volume at indicated time points (**M**) in NSG mice; 1 × 10^7^ cells/mouse. *n* = 4 mice per group. Data in **B**, **D**, **J**, **K**, and **M** are presented as means ± SD. NS, not significant, *P < 0.05, **P < 0.01 versus controls. The underlying data can be found in S1 Data. ERK5, extracellular signal–regulated kinase 5; ERM, Ezrin (Thr567)/Radixin (Thr564)/Moesin (Thr558); HR, hazard ratio; IB, immunoblot; KO, knockout; NS, not significant; NSG, NOD scid gamma; OE, overexpressing; PDCD10, programmed cell death protein 10; p-ERM, phosphorylation of ERM; RhoA, Ras homolog family member A; ROCK1, Rho-associated coiled-coil kinase 1; shRNA, short hairpin RNA; TRIM59, tripartite motif 59; WT, wild-type.

Given our observations that TRIM59 was up-regulated in human breast tumors and that TRIM59 stabilizes PDCD10 protein, we asked whether PDCD10 is essential for breast tumor growth and if the expression of PDCD10 is correlated with TRIM59. As shown in **Fig 6A and 6B**, KO of either *TRIM59* or *PDCD10* suppressed MCF7 xenograft tumor growth to a similar extent. Similar to TRIM59 (**Fig 1E**), PDCD10 expression was correlated with shortened breast cancer patient survival (**Fig 6C**), suggesting a potential pro-oncogenic role of both TRIM59 and PDCD10 in breast cancer.

We next examined how *TRIM59* depletion impinged on the major downstream signaling components of PDCD10. Recent studies have identified several major affected pathways resulting from *PDCD10* loss, including (i) MEKK3/ERK5 signaling and target genes KLF2, KLF4, and NOS3 and ADAMTS protease ADAMTS4 and ADAMTS5 [40,41]; (ii) endothelial-mesenchymal transition (EndoMT) marker S100A4 [42]; and (iii) exocytosis gene ANGPT2 [43]. With quantitative PCR (qPCR), we found that TRIM59 had an inverse correlation with the expression of the majority of PDCD10-regulated genes (**Fig 6D**). At the protein level, we focused on analyzing the phosphorylation status of ERK5, a downstream effector of the PDCD10/MEKK3 signaling that is responsible for activating KLF2/4 and ADAMTS4/5 expression [40, 41]. Following TRIM59 inactivation, we observed a marked increase in phosphorylated ERK5 but not the total ERK5, suggesting that TRIM59 indeed augments PDCD10-mediated signaling and its downstream effectors (**Fig 6E**). Together, our data suggest that TRIM59 modulates PDCD10 protein stability to affect PDCD10-associated downstream signaling.

To test whether PDCD10-ROCK signaling is downstream of TRIM59 but upstream of MLC/ERM to impact breast cancer cells’ contractility (**Fig 4**), we performed functional rescue experiments by using a ROCK inhibitor (Y-27632) or through the lentivirus-mediated stable expression of PDCD10 (**Fig 6F**) in *TRIM59* KO MCF7 cells. Both approaches reduced the appearance of ROCK-mediated membrane blebbing (**Fig 6G**) and attenuated the phosphorylation of ERM induced by *TRIM59* deletion (**Fig 6H**). In MDA-MB-231 cells, depletion of *PDCD10* by shRNA enhanced the phosphorylation of ERM. In MDA-MB-231 cells overexpressing TRIM59 with the resultant suppression of p-ERM, *PDCD10* depletion restored p-ERM to a level that was comparable to WT (**Fig 6I**). Furthermore, re-expression of PDCD10 in *TRIM59* KO cells substantially restored the efficiency of MCF7 cell colony formation (**Fig 6J**), enhanced cell invasion (**Fig 6K**), and rescued the growth of xenograft tumors (**Fig 6L and 6M**). Collectively, these findings suggest that TRIM59 promotes breast cancer growth and migration/invasion through PDCD10-induced suppression of ROCK signaling and subsequent MAT.

### TRIM59 blocks RNFT1-mediated K63 ubiquitination and p62-selective autophagic degradation of PDCD10

**Fig 7:**
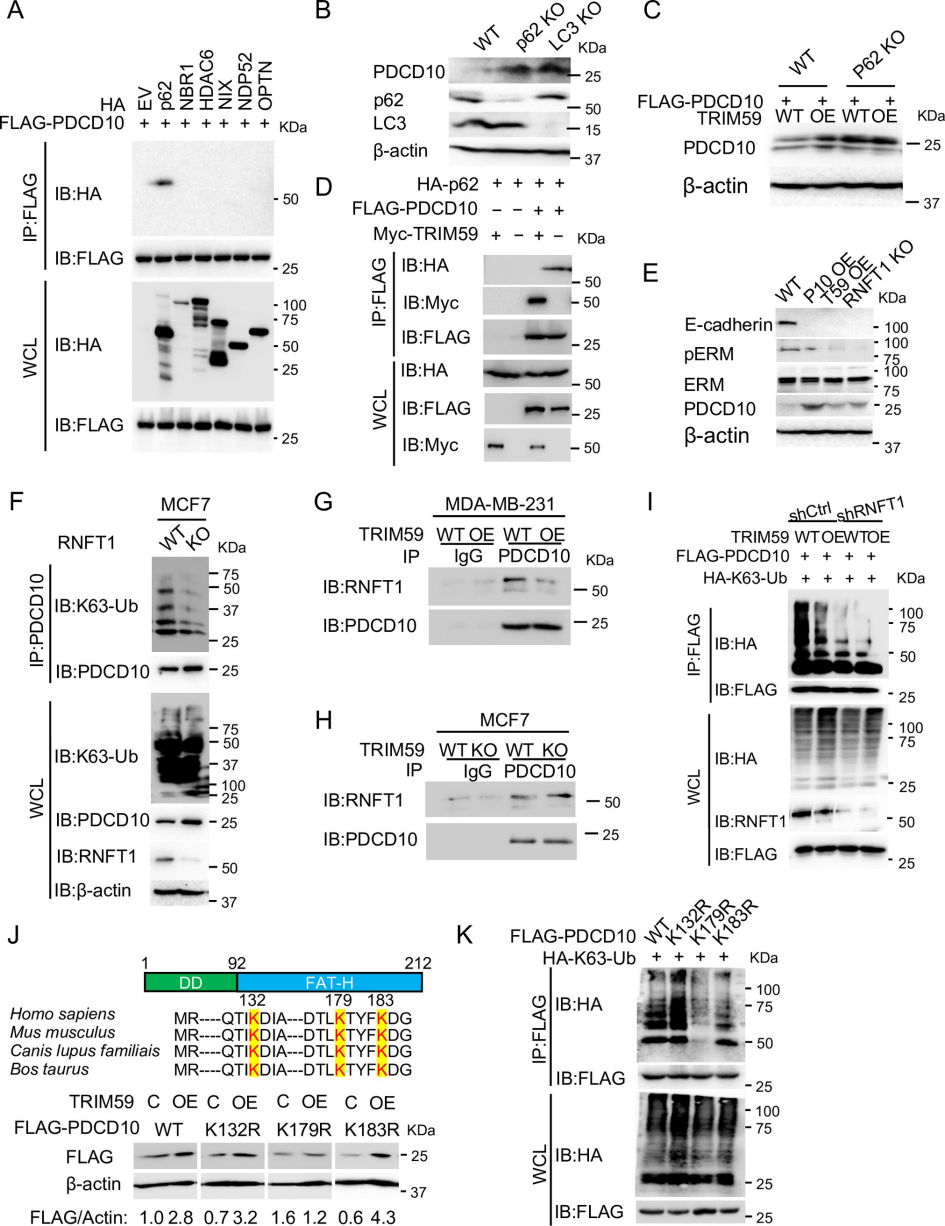
TRIM59 blocks RNFT1-mediated PDCD10 K63 ubiquitination and p62-selective autophagic degradation. (**A**) Co-IP and IB analysis of extracts of HEK293T cells transfected with FLAG-PDCD10, along with expression vectors for cargo receptors HA-p62, HA-NBR1, HA-HDAC6, HA-NIX, HA-NDP52, or HA-OPTN. (**B**) IB analysis of PDCD10, p62, and LC3 expression in WT, p62 KO, and LC3 KO MCF7 cells. (**C**) IB analysis of PDCD10 expression in WT and p62 KO HEK293T cells with or without overexpression of TRIM59 (WT versus TRIM59 OE). (**D**) Co-IP and IB analysis of extracts of HEK293T cells transfected with HA-p62, FLAG-PDCD10, along with empty vector or Myc-TRIM59. (**E**) IB analysis of WT, PDCD10 OE, TRIM59 OE, and *RNFT1* KO MCF7 cells using anti-E-cadherin, anti-p-ERM, anti-ERM, or anti-PDCD10 antibodies. (**F**) Co-IP and IB analysis of extracts of WT or *RNFT1* KO MCF7 cells with indicated antibodies. (**G** and **H**) Co-IP and IB analysis of extracts of WT or TRIM59 OE MDA-MB-231 cells (**G**) and WT or *TRIM59* KO MCF7 cells (**H**) with indicated antibodies and IgG controls. Cells were treated with BafA1 to maintain PDCD10 levels. (**I**) Co-IP and IB analysis of extracts of WT or TRIM59 OE stable HEK293T cells transfected with control shRNA or sh*RNFT1*, along with expression vectors for FLAG-PDCD10, HA-K63. Cells were treated with BafA1 to maintain PDCD10 levels. (**J**) IB analysis of extracts of HEK293T cells transfected with FLAG-PDCD10 WT or mutants (K132R, K179R, and K183R), along with empty vector control (C) or vector for overexpressing (OE) TRIM59. Conserved lysine residues within the FAT-H domain (aa 92–212) were highlighted. The relative expression levels of FLAG-PDCD10 WT or mutants were indicated below the blot images after performing densitometric scanning of blots with subsequent normalization to β-actin. (**K**) Co-IP and IB analysis of extracts of HEK293T cells transfected with FLAG-PDCD10 WT or mutants (K132R, K179R, K183R), along with the expression vector for HA-K63-Ub. BafA1, bafilomycin A1; C, empty vector control; co-IP, co-immunoprecipitation; DD, dimerization domain (aa 1–92); ERM, Ezrin (Thr567)/Radixin (Thr564)/Moesin (Thr558); EV, empty vector; FAT-H, focal adhesion targeting-homology; HA, hemagglutinin; HDAC6, histone deacetylase 6; IB, immunoblot; IgG, immunoglobulin G; IP, immunoprecipitation; KO, knockout; K63-Ub, lysine 63-linked polyubiquitin; LC3, microtubule-associated protein 1A/1B-light chain 3; Myc, avian myelocytomatosis virus oncogene cellular homolog; NBR1, neighbor of BRCA1 gene 1; NDP52, nuclear Domain 10 Protein 52; NIX, BCL2 interacting protein 3 like; OE, overexpressing; OPTN, optineurin; PDCD10, programmed cell death protein 10; p-ERM, phosphorylation of ERM; p62, phosphotyrosine-independent ligand for the Lck SH2 domain of 62 kDa; RNFT1, RING finger and transmembrane domain-containing protein 1; shRNA, short hairpin RNA; TRIM59, tripartite motif 59; WCL, whole cell lysate; WT, wild-type.

Our above results showed that TRIM59 suppressed autophagy-mediated degradation of PDCD10, but it remained unclear what mechanisms TRIM59 might employ to stabilize PDCD10. An E3 ligase typically prevents protein from degradation through three major mechanisms: (1) the recruitment of deubiquitinase [44], (2) facilitating the degradation of another E3 ligase in order to stabilize the target protein [45], and (3) nonproteolytic role of an E3 ligase in the blockade of E3-substrate complex [46]. Emerging evidence shows that different cargo receptors (e.g., p62, neighbor of BRCA1 gene 1 [NBR1], histone deacetylase 6 [HDAC6], BCL2 interacting protein 3 like [NIX], nuclear Domain 10 Protein 52 [NDP52], optineurin [OPTN]) can discriminate and direct specific cargo proteins for the lysosomal degradation during autophagy [47]. Because Ub is known as a signal for selective autophagy-mediated cargo protein degradation recognized by the Ub-associated domain (UBA) of cargo receptors [48,49], we first set out to test if TRIM59 modulates the ubiquitination of PDCD10. We found that ectopically expressed TRIM59 inhibited PDCD10 ubiquitination mediated by either WT Ub or K63-linked Ub (**S5A Fig**). After screening the interaction of PDCD10 with cargo receptors, we identified that PDCD10 specifically engaged p62 but not other receptors (**Fig 7A**). These findings point to the possibility that TRIM59 might suppress the K63 ubiquitination and p62-selective autophagic degradation of PDCD10.

p62 has been demonstrated to interact with LC3 to facilitate the lysosomal degradation of ubiquitinated proteins by autophagy [50]. Upon genetic depletion of p62 or LC3 in MCF7 cells, PDCD10 protein level was profoundly up-regulated compared with WT cells (**Fig 7B**). TRIM59 overexpression was unable to further stabilize the protein level of PDCD10 upon p62 depletion (**Fig 7C**). Next, we hypothesized that TRIM59 might stabilize PDCD10 by blocking the interaction between PDCD10 and p62. Indeed, the interaction of PDCD10 with p62 was abolished after overexpression of TRIM59 (**Fig 7D**). However, we failed to detect a binary interaction between the full-length TRIM59 and p62, although a weak binding was detected between the ΔTM mutant and p62 (**S5B Fig**). Ectopic expression of TRIM59 did not affect the protein level of p62 (**Fig 7D**, whole cell lysate (WCL), IB: HA, lane 1 versus lane 2). These results hint that TRIM59 might block K63 ubiquitination of PDCD10 by exerting an inhibitory effect on a yet-to-be-identified E3 ligase that directly targets PDCD10.

So far, there is no reported E3 ligase for PDCD10. In our yeast two-hybrid screening for potential TRIM59 binders (**S3 Table**), we identified a previously uncharacterized E3 ligase, RNFT1 [23], thus prompting us to speculate that RNFT1 might be the E3 ligase for mediating PDCD10 K63 ubiquitination. Similar to the scenario visualized in either PDCD10 OE or TRIM59 OE cells, we detected a marked increase of PDCD10 protein levels upon depletion of RNFT1 (**Fig 7E and 7F**). Endogenous K63 ubiquitination of PDCD10 was also diminished considerably in RNFT1 KO MCF7 cells compared with WT (**Fig 7F**). To examine if RNFT1 bound to PDCD10 and TRIM59 blocked the interaction between PDCD10 and RNFT1, we performed co-IP experiments in MDA-MB-231 cells (WT versus TRIM59 OE) and MCF7 cells (WT versus TRIM59 KO). We detected the endogenous interaction between RNFT1 and PDCD10 in both breast cancer cell lines but not for IgG controls (**Fig 7G and 7H**; IB: RNFT1, lanes 3 versus 1). TRIM59 OE or TRIM59 KO suppressed or stabilized the PDCD10-RNFT1 interaction, respectively (**Fig 7G and 7H**; IB: RNFT1, lanes 3 versus 4). Following depletion of *RNFT1* in MCF7 cells, we observed a dramatic reduction in K63 ubiquitination of PDCD10. In *RNFT1* KD cells, TRIM59 overexpression was unable to further inhibit the K63 ubiquitination of PDCD10 (**Fig 7I**). Collectively, our results suggest that TRIM59 blocks RNFT1-mediated K63 ubiquitination of PDCD10.

Finally, to pinpoint the potential Ub site within PDCD10, we generated several lysine (K)-to-arginine (R) mutations on conserved key residues (K132, K179, K183) in the C-terminal focal adhesion targeting-homology (FAT-H) domain of PDCD10, which might be involved in the linkage with Ub [51] (**Fig 7J**). We found that, as seen with WT PDCD10, TRIM59 overexpression in HEK293T cells led to the accumulation of the K132R and K183R mutants, but not the K179 mutant (**Fig 7K**). In addition, K63 ubiquitination of PDCD10 was blocked when K179 was mutated (**Fig 7K**). Therefore, our data imply that TRIM59-mediated stabilization of PDCD10 might require K179, a site that could be potentially ubiquitinated by RNFT1.

## Discussion

The TRIM family proteins, most of which have E3 Ub ligase activity, play crucial roles in (i) cancer cell metabolism by regulating adenosine monophosphate (AMP)-activated protein kinase (AMPK) stability [5], (ii) cancer cell proliferation and apoptosis by targeting p53 [4], and (iii) oncogenic transcriptional activation [52]. The study of TRIM proteins in cancer motility (mode of migration and invasion) and metastasis remains largely an uncharted territory. Cancer cells adopt several types of motility modes: (1) mesenchymal type and lamellipodia mode with elongated morphology directed by Rac signals, (2) blebbing and amoeboid mode with small F-actin-rich protrusions regulated by Rho-kinase signaling [12,53], and (3) collective invasion of tumor cell clusters, with leading cells expressing basal epithelial markers such as cytokeratin-14 or p63 [54,55]. In this study, we have uncovered a previously uncharacterized role of TRIM59 in modulating the motility modes and metastasis of cancer cells through coordinated actions of the adhesion molecules and the organization of actin/myosin (actomyosin)-mediated cell contractility. Specifically, *TRIM59*-deleted breast cancer cells favor the amoeboid mode through (i) augmented activation of actomyosin contractile proteins MLC2 and ERM, (ii) excessive formation of E-cadherin-mediated focal adhesion [56], and (iii) the suppression of metastasis-associated Wnt/β-catenin signaling [32–35]. All these factors promote cell–cell adhesion and mesenchymal (or lamellipodia) to amoeboid-like movement transition (MAT), leading to suppressed collective tumor cell migration.

In our effort to apply a yeast two-hybrid screen for the identification of direct targets of TRIM59 in the regulation of cell motility, PDCD10 stood out as a strong candidate. Loss of *TRIM59* or *PDCD10* suppresses tumor growth both in vitro and in vivo, and the amount of both proteins inversely correlates with survival of breast cancer patients. Several lines of evidence have implicated critical roles of PDCD10 in the suppression of ROCK/Rho signaling-mediated phosphorylation of MLC to regulate actomyosin contractility [15–17], whose expression is aberrantly up-regulated in metastatic colon cancer cells [57] and pancreatic adenocarcinomas [58]. In addition, Rho-ROCK activation phosphorylates ERM to regulate cell apoptosis [59]. Thus, regulation of Rho-ROCK signaling by PDCD10 might directly affect the phosphorylation status of MLC and ERM. On the other hand, PDCD10 has been shown to be important for Ste20-like kinase (STK)-mediated phosphorylation of ERM after cells were exposed to reactive oxygen species to protect cells from death under oxidative stress conditions [60]. Multiple reports have shown that ERM, instead of a target of RhoA signaling [59], may function upstream of RhoA in zebrafish [61]. Yet, it is unclear if cancer cells have hijacked different pathways other than the PDCD10-STK-ERM axis for their own benefits to support growth and migration. In this study, we have demonstrated a previously unrecognized role of TRIM59 in breast cancer. TRIM59 directly interacts with and stabilizes PDCD10, leading to suppressed RhoA-mediated MLC and ERM phosphorylation. PDCD10 overexpression or administration of a ROCK inhibitor could reverse *TRIM59* loss-induced contractile phenotypes, thereby accelerating cell migration and invasion. Future genetic and cellular studies of the spatiotemporal organization of PDCD10/STKs complexes under physiological conditions in various cell types will be needed to determine the precise function of PDCD10 in cancer cell migration and metastasis.

In addition, KD or KO of TRIM59 promotes cell apoptosis and cell cycle arrest at G0/G1 phase, as revealed by increased cleaved-caspase3 and Annexin V/7-AAD staining and elevated expression of G1/S transition inhibitors p21 and p27, with concomitant down-regulation of G1/S transition promoter CDK1 in the cell cycle [62] revealed by RPPA analysis. These phenotypes are reminiscent of p53-mediated antitumor activity by inducing cell senescence, cell cycle arrest, and apoptosis [10,63]. However, p53 expression remained largely unaltered in *TRIM59* KD MCF7 cells, thus clearly indicating the existence of a p53-independent mechanism. Notably, proteomic mapping of PDCD10 interactors has identified proteins involved in the regulation of G0/G1-S phase transition of the cell cycle, such as cytoskeletal protein guanine nucleotide-binding protein subunit beta-like protein (GBLP) [14,64]. Further analysis is needed to clarify whether PDCD10 is directly responsible for the function of TRIM59 in cell survival and cell cycle regulation.

Besides the observation of TRIM59 in the regulation of breast cancer cell survival and mode of cancer cell migration and metastasis, our unbiased RPPA study revealed that other critical oncogenic pathways are altered upon *TRIM59* depletion in breast cancer cells. We detected a significant reduction in VEGFR2 protein expression upon KD or KO of *TRIM59* in MCF7 cells. Interestingly, one recent study has shown that PDCD10 colocalizes with VEGFR2. Loss of *PDCD10* decreased the stability of VEGFR2 protein [65], hinting that TRIM59 might stabilize VEGFR2 protein through PDCD10 to augment VEGF signaling and tumor angiogenesis [65]. Moreover, the metabolic states of cancer cells determine their fates in a nutrient-poor environment [66]. *TRIM59* depletion in breast cancer cells could further impinge on genes and pathways involved in cancer metabolism: (1) down-regulation of metabolism-related oncogenes such as c-MYC and IGF1R [67,68]; (2) up-regulation of tumor suppressors INPP4b, a PtdIns(3,4,5) P3 phosphatase that modulates membrane lipid metabolism and inhibits protein kinase B (AKT) activation [69]. Therefore, our data suggest a multifaceted function of TRIM59 in regulating breast cancer angiogenesis and metabolism, which will be further subjected to mechanistic dissection in follow-on studies in the near future.

**Fig 8:**
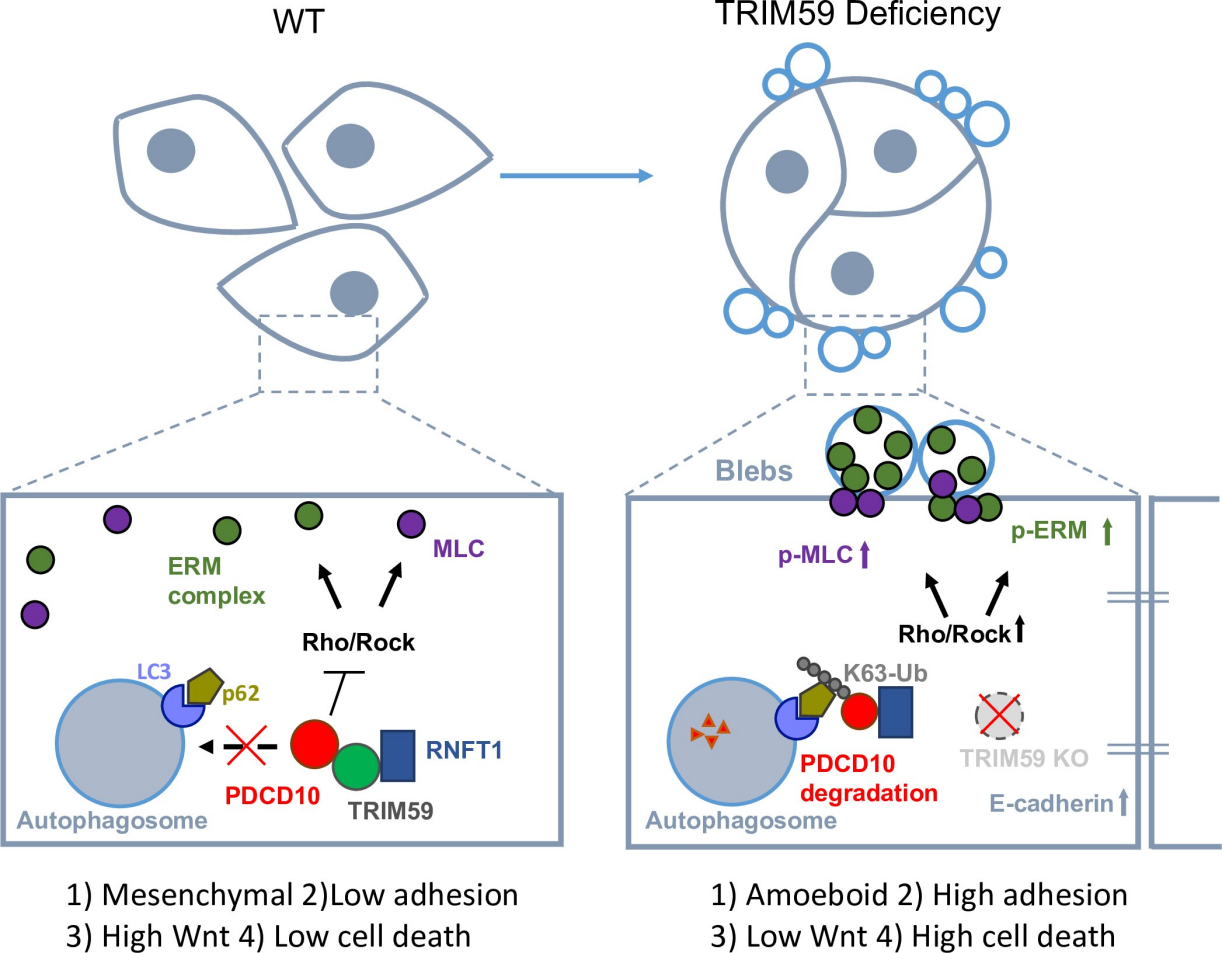
TRIM59 depletion attenuates breast cancer cell survival and metastasis. TRIM59 is essential for breast cancer cell mesenchymal movement and cell survival by maintaining low cell adhesion and high Wnt signaling of breast cancer cells. TRIM59 stabilizes PDCD10 by inhibiting K63 ubiquitination induced by RNFT1 at the lysine 179 site, and subsequently suppresses p62-selective autophagy degradation. *TRIM59* deficiency facilitates PDCD10 degradation to overcome its inhibitory effect on Rho/ROCK signaling, thereby causing hyperactivated MLC and ERM and mesenchymal to amoeboid transition (MAT). *TRIM59* deficiency also promotes excessive E-cadherin expression and reduces β-catenin expression, leading to high cell adhesion, low Wnt signaling, and high cell death to ultimately curtail tumor formation and metastasis. ERM, Ezrin (Thr567)/Radixin (Thr564)/Moesin (Thr558); KO, knockout; K63-Ub, lysine 63-linked polyubiquitin; LC3, microtubule-associated protein 1A/1B-light chain 3; MAT, mesenchymal to amoeboid transition; MLC, myosin light chain; PDCD10, programmed cell death protein 10; p-ERM, phosphorylation of ERM; p-MLC, phosphorylation of MLC; p62, phosphotyrosine-independent ligand for the Lck SH2 domain of 62 kDa; Rho/ROCK, Ras homolog/Rho-associated coiled-coil kinase; RNFT1, RING finger and transmembrane domain-containing protein 1; TRIM59, tripartite motif 59; Ub, ubiquitin; WT, wild-type.

In summary, our study has unveiled TRIM59 as an essential tumor-promoting factor that facilitates breast cancer growth and metastasis through modulating PDCD10-associated signaling pathways, as well as other oncogenic pathways. Loss of *TRIM59* sensitizes PDCD10 for autophagy-mediated degradation and subsequently induces robust ROCK activity to promote cell adhesion and suppress mesenchymal or lamellipodia movement (**Fig 8**). Hence, a therapeutic intervention that interrupts the functional interplays between TRIM59 and PDCD10 might provide a promising strategy to treat breast cancer and CCM.

## Materials and methods

### Ethics statement

The use of human cancer cell lines was in accordance with institutional guidelines on human cell research and the approved protocol (IBC2015-132) by the Institutional Review Board. Animal experiments in this study were approved and carried out in accordance with the protocol (2015-0342-IBT) provided by the Institutional Animal Care and Use Committee (IACUC) at Institute of Biosciences and Technology, College of Medicine, Texas A&M University. IACUC uses the National Institute of Health (NIH) Guide for the Care and Use of Laboratory Animals, which is based on the United States Government Principles for Utilization and Care of Vertebrate Animals Used in Testing, Research, and Training.

### Cell lines, antibodies, and reagent

Breast cancer cell lines MDA-MB-436 (HTB-130), MDA-MB-231 (HTB-26), MDA-MB-468 (HTB-132), MDA-MB-175-VII (HTB-25), CAMA-1 (HTB-21), MDA-MB-453 (HTB-131), MDA-MB-361 (HTB-27), MCF7 (HTB-22), normal mammary epithelial cell line MCF10A (CRL-10317) cells, and HEK293T cells (CRL-3216) were purchased from American Type Culture Collection (ATCC, Manassas, VA) and maintained in DMEM or RPMI-1640 medium with 10% fetal bovine serum (FBS) according to culture instructions provided by ATCC. Cell lysate was collected by RIPA buffer (10 mM Tris, pH 7.2, 0.1% SDS, 0.1% v/w Triton X-100, 0.1% deoxycholate, 5 mM EDTA) with protease/phosphatase inhibitor cocktail (#5872, Cell Signaling Technology, Danvers, MA). Protein concentration was determined by BCA assay using BCA Protein Assay Reagent (#23225, Thermo Fisher Scientific, Waltham, MA). A total of 30–60 μg of cell lysate was subjected to western blot analysis.

Antibodies used in western blot and immunoprecipitation include primary antibodies against PDCD10 (#sc-365587, for immunoprecipitation and immunofluorescent staining), p53 (#sc-126), and total ERM antibody (#sc-22807) were purchased from Santa Cruz Biotechnology (Dallas, TX). PDCD10 antibody (#ABN-1016, for IB analysis) was purchased from Millipore (Burlington, MA). TRIM59 antibody (#NBP-82625) was purchased from Novus Biologicals (Littleton, CO) and TRIM59 antibody (#ab69639) was purchased from Abcam (Cambridge, United Kingdom). RNFT1 antibody (#PA5-48913) was purchased from Thermo Fisher Scientific (Waltham, MA). Cleaved-Caspase3 (#9661), Ki-67 (#9027), phosphorylated (p)-SAPK/JNK (#9251), p-p38 MAPK (#4511), p-p44/p42 (#9101), p-ERK5 (#3371), total ERK5 (#3552), p-Ezrin (Thr567)/Radixin (Thr564)/Moesin (Thr558) antibody (#3726), Epithelial-Mesenchymal Transition (EMT) Antibody Sampler Kit (#9782), Cadherin-Catenin Antibody Sampler Kit (#9961), and p-myosin light chain 2 (Ser19) (#3671) were purchased from Cell Signaling Technology (Danvers, MA). anti-FLAG M2 affinity gel (#A2220), anti-FLAG M2-peroxidase (HRP) antibody (#A8592), and anti-HA−HRP antibody (#H6533) were purchased from Sigma-Aldrich (St. Louis, MO).

### Patient samples and gene expression profiling analysis of human breast cancer

Fresh samples of human breast cancer and paired adjacent normal tissues (paired adjacent normal versus cancerous tissues, *n* = 90), as well as metastatic cancer and paired primary cancer tissues (paired primary versus metastatic samples, *n* = 19) were obtained during surgery at Sir Run Run Shaw Hospital of Zhejiang University. All samples were collected with patients’ informed consent and approved by Institutional Review Board. For assessing the correlation between TRIM59 IHC scores and clinical stages of breast cancer, a total of 170-spot, paraffin-embedded tissue array chips (HBre-Duc170Sur-01), including 90 paired breast tumor and normal tissues, 70 tumor tissues, and 10 normal tissues with 10 to 13.5 years of follow-up information, were purchased from Shanghai Outdo Biotech (Shanghai, China). Please note that we deleted nine cases (J07A0938, J07A0942, J07A0944, J07A0946, J07A0958, J07A0973, J07A0999, J07A1044, J07A1131) based on the 154 cases listed in S1 Table prior to the multivariate analysis, as N and AJCC stage information was absent in J07A0938, J07A0958, J07A0973, and J07A1044, and PR and/or ER and/or HER2 information was absent in J07A0942, J07A0944, J07A0946, J07A0999, and J07A1131. Detailed clinical features of breast cancer samples are summarized in **S1**and **S2 Tables**. Gene expression profiling (GEP) analysis of TRIM59 protein expression (in both primary and metastatic breast cancer samples) was performed on datasets from Gene Expression Omnibus (GEO, https://www.ncbi.nlm.nih.gov/geo/) GSE30480. Specifically, we included 14 primary breast tumors and 6 metastatic lymph node samples. Statistical differences among multiple groups were analyzed with one-way ANOVA test. Significant differences were determined when the *P* value was less than 0.05.

### Immunoprecipitation and IB analysis

For immunoprecipitation experiments, whole cell extracts obtained 24 hours after transfection were lysed in low salt lysis buffer (50 mM Tris, pH 7.5; 150 mM NaCl; 1% Triton-X; 5 mM EDTA; 10% [v/v] glycerol with protease/phosphatase inhibitor cocktail) or RIPA buffer and shaken on ice for 15 minutes. Whole cell lysates were incubated for 4 hours at 4°C with the indicated antibodies and protein A/G beads (#20421, Thermo Fisher Scientific, Waltham, MA) or with anti-FLAG (#A2220, Sigma-Aldrich, St. Louis, MO) or anti-HA (#A2095, Sigma-Aldrich, St. Louis, MO) agarose gels for FLAG- OR HA-tagged protein pull-down. After incubation, beads were washed five times with 1 mL low salt lysis buffer or RIPA buffer, boiled with 4× SDS loading buffer, and subjected to SDS-PAGE. Proteins were transferred to nitrocellulose membranes (Bio-Rad, Hercules, CA). Membranes were blocked in 5% dry milk/TBST for 1 hour and incubated using indicated antibodies. IB analysis was developed using the Luminata Western HRP Chemiluminescence Substrates (#WBLUF0500, Millipore, Burlington, MA) and ChemiDoc XRS+ System with Image Lab (Bio-Rad, Hercules, CA).

### IHC and immunofluorescent assay

The TRIM59 levels in breast tumors and normal breast tissues were evaluated by IHC using an anti-TRIM59 antibody on paraffin-embedded tissue samples or commercial tissue arrays (Shanghai Outdo Biotech, Shanghai, China) as previously described [70]. Briefly, sections were dewaxed, hydrated, and washed. After neutralization of endogenous peroxidase and microwave antigen retrieval, slides were preincubated with blocking serum and then incubated for 1 hour at 37°C with indicated antibodies. Subsequently, the sections were serially rinsed, incubated with the second antibody, and treated with HRP-conjugated streptavidin. Reaction products were visualized with 3, 3-diaminobenzidine tetrahydrochloride and counterstained with hematoxylin. TRIM59 IHC scores were calculated from TRIM59 IHC staining intensity multiplied by positive staining rate. Staining intensity is generally divided into four levels: 0 indicates negative staining, 0.5 indicates weakly positive, 1 indicates staining intensity is light yellow or light brown, and 2 indicates staining intensity is yellow or brown. The ratio of the number of positive cells to the total number of such cells in the tissue was defined as the positive staining rate.

The immunofluorescence assays and confocal microscopy were conducted as described previously [71]. HEK293T, MCF7, or MDA-MB-231 cells were grown on the glass-bottom dish (MatTek, Ashland, MA) in complete medium. Cells were then fixed in 4% paraformaldehyde solution in PBS at room temperature for 15 minutes and permeabilized at room temperature with 0.1% Triton X-100/PBS. Subsequently, cells were blocked in 5% normal goat serum (NGS) for 1 hour at RT, and then incubated in indicated primary antibodies in SignalStain antibody diluent (#8112, Cell Signaling Technology, Danvers, MA) for 16 hours at 4°C. Cells were then washed three times with PBS and incubated with the secondary fluorescent antibody (1:400 dilution) for 1 hour. The nucleus was then labeled with DAPI for 5 minutes in the dark and then followed by three washes in PBS. For monitoring autophagosome formation, cells were transfected with expression vector for GFP-LC3 (#psetz-gfplc3, Invivogen, San Diego, CA). Samples were then visualized using Nikon Eclipse Ti-E microscope. All acquired images were analyzed and the correlation coefficient (r) of pixel intensity values was extracted by using the Nikon NIS-Elements AR package or the ImageJ (NIH) software.

### Yeast two-hybrid screening

Yeast two-hybrid screening was carried out with Matchmaker Gold Yeast Two-Hybrid System (#630489, Clontech Laboratories, Mountain View, CA) using the AH109 yeast strain, as previously described [72]. To construct a bait plasmid, full-length TRIM59 was cloned in-frame into the GAL4 DNA-binding domain of pGBKT7. A normalized human universal Matchmaker cDNA library (#630481, Clontech Laboratories, Mountain View, CA) was used to screen about 1 × 10^6^ clones. Positive clones were picked up and library plasmids were recovered and expanded in *Escherichia coli*. The inserted cDNAs were sequenced and then characterized with the BLAST program.

### Lentivirus production and transduction

Bacterial strains containing the plasmids for GIPZ lentiviral shRNA targeting *TRIM59*, *PDCD10*, or *RNFT1* were purchased from Dharmacon. Plasmid purification was conducted by using the QIAGEN Plasmid Maxi kit (#12162, QIAGEN, Hilden, Germany).

HEK293T cells were used to produce lentivirus. Co-transfection of the lentiviral plasmids, pMD2.G (#12259, Addgene, Cambridge, MA) and psPAX2 (#12260, Addgene, Cambridge, MA), was done by CalPhos Mammalian Transfection Kit (#631312, Clontech Laboratories, Mountain View, CA). Forty-eight and seventy-two hours after transfection, virus-containing cell culture supernatant was collected and passed through a 0.45-μm filter for virus preparation. HEK293T, MCF7, or MDA-MB-231 cells were seeded at 70% confluence 1 day before infection. A total of 5 mL of virus-containing cell culture supernatant with 8 μg/mL polybrene (#TR-1003-G, Millipore, Burlington, MA) was added to each 10-cm dish for 3 hours before adding 5 mL of complete DMEM containing 10% FBS. Virus infection was repeated 24 hours later. Between 48 and 72 hours after the first infection, MCF7 or MDA-MB-231 cells were harvested and seeded in 96-well plates at 1 cell/well for single colony selection. *TRIM59* KD efficiency was assessed by quantitative PCR.

### RPPA analysis

Cells were washed twice in ice-cold PBS, lysed in 500 μL lysis buffer (1% Triton X-100, 50 mM HEPES, pH 7.4, 150 mM NaCl, 1.5 mM MgCl_2_, 1 mM EGTA, 100 mM NaF, 10 mM Na pyrophosphate, 1 mM Na_3_VO_4_, and 10% glycerol, with freshly added protease/phosphatase inhibitor cocktail) for 20 minutes on ice, with occasional shaking every 5 minutes, and centrifuged at 14,000 rpm for 10 minutes at 4 ^o^**C** to collect supernatant. Protein concentration was determined by BCA assay and adjusted to 1–1.5 mg/mL. Cell lysate was mixed with 4×SDS sample buffer without bromophenol blue (40% glycerol, 8% SDS, 0.25 M Tris-HCl, pH 6.8, with freshly added 2-mercaptoethanol at 1/10 of the volume). A 100-μL sample for each replicate was sent for analysis. RPPA assay was done at the RPPA Core Facility of M.D. Anderson Cancer Center (Houston, TX). A full list of antibodies (Ab List_218) used in this study was described as follows: https://www.mdanderson.org/research/research-resources/core-facilities/functional-proteomics-rppa-core/antibody-information-and-protocols.html.

### Construction of sgRNA/Cas9 LentiCRISPR

We designed human single guide RNAs (sgRNAs) as previously described [73], using the online CRISPR design tool (crispr.mit.edu), by inputting targeted exon sequence. *TRIM59*, *PDCD10*, *p62*, *LC3*, or *RNFT1* sgRNA was designed and cloned into the BsmB1 site of lentiCRISPR vector containing Cas9-P2A-puromycin and was verified by sequencing analysis. The sgRNA-containing plasmids were transfected into HEK293T cells with pMD2.G and psPAX2. After 2 days, the virus-containing medium was subjected to ultracentrifugation (20,000*g* at 4°C for 2 hours) and frozen at −80°C. HEK293T, MCF7, or MDA-MB-231 cells were transduced with control sgRNA or *TRIM59*-, *PDCD10*-sgRNA–, or *RNFT1*-sgRNA–containing lentiCRISPR viruses. Transduced cells were selected in the presence of puromycin (#A1113803, ThermoFisher, Waltham, MA) for 48 hours and subjected to ligand stimulation or viral infection. The primers used for sgRNA are as follows:

*TRIM59 sgRNA*: 5′-caccgCCAGCATGTACAGATCTTGAAA-3′
*PDCD10 sgRNA*: 5′-caccgGGGCATAGAAACCATGGATG-3′
*RNFT1 sgRNA*: 5′-caccgTCATGACCAGAAGCGGACGG-3′
*LC3 sgRNA*: 5′-caccgACCTCCTTACAGCGGTCGGC-3′
*p62 sgRNA*: 5′-caccgCACCGTGAAGGCCTACCTTC-3′

### Wound healing assay

A total of 1 × 10^6^ MCF7 or MDA-MB-231 cells were plated and cultured in 6-well plates until they reached 100% confluence, followed by overnight starvation in medium containing 0.5% FBS. Starved cells were pretreated with mitomycin C (#M4287, Sigma-Aldrich, St. Louis, MO) at 20 μg/mL for 4 hours. A wound was made in the confluent cell layer by scratching wells in vertical and horizontal directions. Cells were then washed with PBS twice and migration was induced by complete medium containing 10% FBS. Cell migration was checked every 12 hours until the wound healed in one of the samples. Migration was stopped by removing growth medium and fixing cells in 4% paraformaldehyde.

### Transwell invasion assay

Growth factor–reduced Matrigel (#356234, BD Biosciences, San Jose, CA) was mixed with serum-free MEM Eagle medium at 1:4. A total of 100 μL of the mixture was added to the bottom of cell culture inserts with 8-μm pore size. Cell culture inserts were incubated at 37**°**C for 1 hour.

Cells were serum starved overnight in DMEM containing 0.5% FBS. Starved cells were pretreated with mitomycin C at 20 μg/mL for 4 hours. Starved cells were harvested and resuspended at 1 × 10^5^ cells/mL in DMEM containing 0.5% FBS. A total of 100 μL of cell suspension was seeded into a cell culture insert with Matrigel at the bottom. A total of 500 μL of complete DMEM containing 10% FBS was added to the lower chamber. Ten percent FBS served as chemoattractant. Cell invasion was checked every 12 hours until a significant population of cells invaded through the 8-μm pore on the cell culture insert (48 hours in most experiments). Noninvasive cells were removed from the top of the filter with a cotton swab. Cells at the bottom of the insert were fixed by immersing cell culture inserts in 4% paraformaldehyde for 15 minutes at room temperature. Cell culture inserts were then washed in PBS. Invaded cells were stained with 0.05% crystal violet in distilled water for 30 minutes. After two washes in PBS, invaded cells were visualized under an Olympus IX70 microscope, and three randomly chosen fields per membrane were photographed and quantitated using the Fiji ImageJ software [74].

### Three-dimensional cell culture

Three-dimensional culture of MCF7 cells was performed as described previously [75]. Briefly, prechilled 24-well plates were coated with a thin layer of BD Matrigel Basement Membrane Matrix and incubated for 30 minutes at 37°C. A total of 2 × 10^3^ cells/well of control shRNA MCF7 or TRIM59 shRNA-MCF7 stable cells suspended in diluted Matrigel (1:4 dilution in RPMI1640 medium with 10% FBS) were added into the well and incubated for 30 minutes at 37°C. A total of 500 μL of culture medium was then added and culture was maintained for 10 days, with medium changed every 2 days. Colony formation was then photographed and quantified.

### qPCR analysis

Total RNA was isolated from cells or tissues, and first-strand cDNA was generated from total RNA using oligo-dT primers and SuperScript III Reverse Transcriptase (#18080093, Thermo Fisher Scientific, Waltham, MA). qPCR was performed using specific primers and the ABI Prism 7000 analyzer (Applied Biosystems, Foster City, CA) with the PowerUp SYBR Green Master Mix (#A25741, Thermo Fisher Scientific, Waltham, MA). Target gene expression values were normalized to human *GAPDH*. The primers used for qPCR are as follows:

*GAPDH*-Fwd: 5′-TCAAGAAGGTGGTGAAGCAG-3′
*GAPDH*-Rev: 5′-GAGGGGAGATTCAGTGTGGT-3′
*MYC*-Fwd: 5′-GGCTCCTGGCAAAAGGTCA-3′
*MYC*-Rev: 5′-CTGCGTAGTTGTGCTGATGT-3′
*AXIN2*-Fwd: 5′-CAACACCAGGCGGAACGAA-3′
*AXIN2*-Rev: 5′-GCCCAATAAGGAGTGTAAGGACT-3′
*S100A4*-Fwd: 5′-GATGAGCAACTTGGACAGCAA-3′
*S100A4*-Rev: 5′-CTGGGCTGCTTATCTGGGAAG-3′
*NOS3*-Fwd: 5′-TGATGGCGAAGCGAGTGAAG-3′
*NOS3*-Rev: 5′-ACTCATCCATACACAGGACCC-3′
*KLF2*-Fwd: 5′-TTCGGTCTCTTCGACGACG-3′
*KLF2*-Rev: 5′-TGCGAACTCTTGGTGTAGGTC-3′
*KLF4-*Fwd: 5′-CCCACATGAAGCGACTTCCC-3′
*KLF4-*Rev: 5′-CAGGTCCAGGAGATCGTTGAA-3′
*ADAMTS4-Fwd*: *5′-GAGGAGGAGATCGTGTTTCCA-3′*
*ADAMTS4-Rev*: *5′-CCAGCTCTAGTAGCAGCGTC-3′*
*ADAMTS5-Fwd*: *5′-GAACATCGACCAACTCTACTCCG-3′*
*ADAMTS5-Rev*: *5′-CAATGCCCACCGAACCATCT-3′*
*ANGPT2-Fwd*: *5′-AACTTTCGGAAGAGCATGGAC-3′*
*ANGPT2-Rev*: *5′-CGAGTCATCGTATTCGAGCGG-3′*
*TRIM59-Fwd*: *5′-AAGATCCTCGTGTACTGCCAT-3′*
*TRIM59-Rev*: *5′-CAATGCCAGTTGGAGCAATTTC-3′*
*PDCD10-Fwd*: *5′-GCCCCTCTATGCAGTCATGTA-3′*
*PDCD10-Rev*: *5′-AGCCTTGATGAAAGCGGCTC-3′*
*RNFT1-Fwd*: *5′-CCTGAAGCAAAGACATCTGGG-3′*
*RNFT1-Rev*: *5′-ACTGTGCAGTTGGCTACGATT-3′*

### Xenograft

Orthotopic mammary fat-pad injection of 8-week-old female NOD.Cg-Prkdc<scid> Il2rg<tm1Wjl>/SzJ (NOD scid gamma, NSG) mice (#005557, The Jackson Laboratory, Bar Harbor, ME) with MCF7 or MDA-MB-231 cells was performed. Briefly, mice were anesthetized and an abdominal incision was made to expose the mammary gland. A total of 1–10 × 10^6^ cells in 50 μL RPMI1640 with 10% FBS were mixed with an equal volume of pathogen-free BD Matrigel Basement Membrane Matrix and injected into the mammary fat-pad. Tumor growth was monitored and measured with a digital caliper, and the tumor volume in mm^3^ is calculated by the formula: volume = (width)^2^ × length/2. At 12 weeks, mice were euthanized and necropsied to remove tumors and organs potentially containing metastatic foci for formalin fixation, paraffin embedding, and tissue analysis.

### Breast cancer metastasis animal model

MCF7 and MDA-MB-231 cells were transduced with a lentivirus containing pLenti-CMV-Luciferase-IRES-GFP, followed by lentiviral transduction for the overexpression or CRISPR/Cas9-mediated disruption of *TRIM59* (empty vector or control sgRNA as negative control). Six-week-old NSG mice were used for the orthotopic injection of breast cancer cells (MCF7, 10^7^ cells/mouse; MDA-MB-231, 2 × 10^6^ cells/mouse) into mammary fat-pad. D-Luciferin (15 mg/mL) 150 mg/kg was injected intraperitoneally into tumor-bearing mice 10 minutes before imaging. The growth and metastasis of the tumors were monitored by weekly bioluminescence imaging using the IVIS imaging system (Xenogen, Waltham, MA). End point assays were conducted 10 or 14 weeks after the inoculation unless the animal euthanasia was required because of significant morbidity.

### Nuclear and cytoplasmic extraction

Nuclear and cytoplasmic fractions were isolated using NE-PER Nuclear and Cytoplasmic Extraction Reagents (#78833, Thermo Fisher Scientific, Waltham, MA). A total of 10^7^ MCF7 cells were harvested with trypsin-EDTA and washed with PBS. A 50-μL cell pellet was resuspended by vigorous vortexing in 500 μL ice-cold CER I solution and incubated on ice for 15 seconds. A total of 27.5 μL ice-cold CER II solution was added and mixed followed by 1 minute of incubation on ice and was then centrifuged for 5 minutes at 16,000*g*. The supernatant fraction containing the cytoplasmic extract was transferred to a clean prechilled tube until use. The insoluble pellet fraction containing the nuclei was suspended in 250 μL ice-cold NER, followed by continuous vortexing for 15 seconds every 10 minutes for 40 minutes. The mixture was then centrifuged at 16,000*g* for 10 minutes. The supernatant fraction containing the nuclear extract was transferred to a clean prechilled tube until use.

### Proliferation and apoptosis assays

Cell proliferation was determined by MTS assay. MCF7 or MDA-MB-231 cells were seeded into 96-well plates in 100 μL of culture medium and cultured for the indicated time. Ten microliters per well of MTS (#G3582, Promega, Madison, WI) was added into each well for a 2-hour incubation at 37°C in a humidified, 5% CO_2_ atmosphere. The absorbance was measured using a model ELX800 Micro Plate Reader (Bio-Tek Instruments, Winooski, VT) at 490 nm then the proliferation was calculated. Annexin V and 7-AAD staining kit (#559763, BD Biosciences, San Jose, CA) was used to detect apoptotic cells. WT, *TRIM59* KD, or *TRIM59* KO MCF7 cells were harvested with trypsin-EDTA and washed with PBS twice. Cells were then resuspended in 1× binding buffer at a concentration of 10^6^ cells/mL. A total of 5 μL Annexin V and 5 μL 7-AAD was added to 100 μL of the cell solution and incubated at RT for 15 minutes in the dark. Stained cells were washed and resuspended in 400 μL of 1× binding buffer and analyzed by flow cytometry.

### Colony formation assay

The colony formation assay was used to reflect anchorage-independent cell growth. About 1 × 10^4^ MCF7 or MDA-MB-231 cells suspended in 1 mL of 0.33% agarose 1640 medium containing 10% FBS were plated in 12-well plates on the top of existing 0.5% bottom agarose with the same medium. The medium was replenished every 3–5 days, and colonies that grew beyond 50 μm in diameter after 2–3 weeks were scored as countable.

### Cell cycle assay

A total of 5 × 10^5^ WT, *TRIM59* KD, or *TRIM59* KO MCF7 cells per well were seeded into 6-well plates and allowed to attach overnight. The culture media were replaced with fresh complete 1640 containing 2 mM thymidine and cultured for 18 hours; after washing with PBS, the block was released by incubation in fresh complete 1640 for 10 hours. Cells were blocked again with fresh complete 1640 containing 2 mM thymidine and cultured for 18 hours; after washing with PBS, the block was released by incubation in fresh complete 1640 for 12 hours. Cells were collected and permeabilized with precooled 75% ethanol at 4°C overnight. The next day, the cells were washed with PBS and incubated with 0.5% PI (#556463, BD Biosciences, San Jose, CA) in the dark for 30 minutes. DNA content was detected by flow cytometry. The data were analyzed with ModFit software.

### TCGA data analysis

Gene expression data were downloaded from the TCGA data portal (https://portal.gdc.cancer.gov/). The normal sample was extracted from the same adjacent non-tumorigenic tissue in the same patient. Comparison of tumor and paired adjacent normal samples is used in TCGA data analysis [76], as described in our previous studies [77]. Genes were considered differentially expressed if the fold change (FC) is >1.5 and *t* test *P* value is <0.05.

### Statistics

Descriptive statistics, including means, standard deviations, medians, and ranges, were computed for each group and analyzed with Student *t* test or for multiple comparisons, with ANOVA. Data were presented as mean ± standard error. The sample size for each experiment, *n*, was included in the results section and the associated figure legend. All analyses were performed with GraphPad Prism 5 (GraphPad Software, La Jolla, CA). *P* values <0.05 were considered significant. Phosphorylation levels of Ezrin in BRCA were obtained from CPTAC [31]. We calculated the Spearman correlation between TRIM59 expression and the phospho-Ezrin (S148, S366) status in BRCA and used Spearman correlation coefficient, |Rs| > 0.2, as statistical significance. We used the Kaplan–Meier method to calculate survival curves and a log-rank test to check whether gene levels were significantly associated with overall patient survival. Patient survival curves based on the expression of PDCD10 (Affymetrix ID: 210907_s_at) were analyzed according to an online survival analysis tool (kmplotter.com), using breast cancer microarray data [78]. Multivariate analysis of prognostic factors was performed by a Cox (proportional hazards) regression model, including TRIM59 levels or clinical information (cancer staging and grading).

## Supporting information

S1 Fig TRIM59 expression in normal tissues and malignancies. **Related to Fig 1.** (**A**) Fold change of TRIM59 expression across 12 cancer types compared with their paired controls (obtained from adjacent noncancerous tissues), based on TCGA dataset analysis. (**B** and **C**) mRNA expression profiles of *TRIM59* in human (**B**) and mouse (**C**) tissues. (**D**) Quantitative RT-PCR of *TRIM59* mRNA expression in human breast cancer cell lines. (**E**-**G**) TRIM59 IHC scores in subtypes of breast cancer based on HER and ER status. (**H**) Multivariate overall survival analysis of patient by using a Cox proportion hazards model. (**I**) Kaplan–Meier survival curves for Grade II or above breast cancer patients. Patients were divided into two groups according to TRIM59 staining scores (Low, with a score less than or equal to 0.75; High, with a score greater than 0.75). The underlying data can be found in S1 Data. ER, estrogen receptor; HER, human epidermal growth factor receptor 2; IHC, immunohistochemistry; RT-PCR, reverse transcription polymerase chain reaction; TCGA, the Cancer Genome Atlas; TRIM59, tripartite motif 59.
(TIF)

S2 Fig Validation of TRIM59 antibodies and RPPA analysis in *TRIM59*-depleted MCF7 cells. **Related to Fig 3.** (**A**) IB analysis of TRIM59 expression in MCF10A, MCF7, and MDA-MB-231 using two TRIM59 antibodies (Novus and Abcam). Purified TRIM59 protein was used as positive control (S2A, left). (**B**) TRIM59 mRNA expression levels in MCF7 and MDA-MB-231 cells revealed by RNA sequencing dataset analysis (GEO database: #GSE54326). *n* = 3. Data are presented as means ± SD. *P < 0.05. (**C**) RPPA analysis of alterations in protein expression levels or phosphorylation in Control, sh*TRIM59*, and *TRIM59* KO MCF7 cells. *n* = 3. The underlying data can be found in S1 Data. IB, immunoblot; RPPA, reverse phase protein array; TRIM59, tripartite motif 59.
(TIF)

S3 Fig TRIM59 promotes Wnt/β-catenin signaling. **Related to Fig 4.** (**A**) Representative immunochemistry staining of β-catenin in tissue sections from xenograft tumors of *TRIM59* KO MCF7 cells or TRIM59 OE MDA-MB-231 cells compared with control cells. Scale bars: 200 μm. (**B**) qPCR quantification of the Wnt signaling pathway downstream gene *AXIN2* in WT and *TRIM59* KO MCF7 cells. *n* = 4. Data are presented as means ± SD. ***P < 0.001 versus WT. The underlying data can be found in S1 Data. KO, knockout; OE, overexpressed; qPCR, quantitative polymerase chain reaction; TRIM59, tripartite motif 59; WT, wild-type.
(TIF)

S4 Fig TRIM59 modulates protein stability of PDCD10 through autophagy pathways. **Related to Fig 5.** (**A**) IB analysis of endogenous TRIM59 expression in MCF7 cells expressing two different shRNAs targeting *TRIM59* compared with scramble shRNA (shControl). (**B**) IB analysis of endogenous PDCD10 expression in MCF7 cells expressing sh*TRIM59*-1 or sh*TRIM59*-2 compared with shControl. MCF7 cells expressing sh*PDCD10*-1 or sh*PDCD10*-2 targeted to PDCD10 were used as control. (**C**) IB analysis of PDCD10 in WT MCF7 or TRIM59 KD MCF7 cells treated with DMSO, BafA1, 3-MA, or MG132 for 6 hours. (D) IB analysis of PDCD10 protein levels in WT or TRIM59 OE MDA-MB-231 cells treated with DMSO or BafA1 for 12 hours. Quantitative comparisons of PDCD10 levels were analyzed by densitometric scanning of blots and normalized to β-actin. (**E**) Co-IP and IB analysis of HEK293T cells transfected with FLAG-p53, along with HA-tagged Ub^WT^, followed by MG132 treatment (0, 6, or 12 hours). (**F**) Representative confocal images of WT or *TRIM59* KO HEK293T cells transfected with GFP-LC3. BafA1, bafilomycin A1; co-IP, co-immunoprecipitation; GFP, green fluorescent protein; HA, hemagglutinin; IB, immunoblot; KD, knockdown; KO, knockout; LC3, microtubule-associated protein 1A/1B-light chain 3; OE, overexpressed; PDCD10, programmed cell death protein 10; shRNA, short hairpin RNA; TRIM59, tripartite motif 59; Ub^WT^, ubiquitin WT; WT, wild-type; 3-MA, 3-methyladenine.
(TIF)

S5 Fig TRIM59 suppresses K63 ubiquitination of PDCD10. Related to Fig 7. (**A**) HEK293T cells were transfected with FLAG-PDCD10 and HA-Ub (WT) or its mutants, along with Myc-TRIM59 or empty vector, in the presence of BafA1. Whole-cell lysates were immunoprecipitated with anti-FLAG beads and immunoblotted with indicated antibodies. (**B**) Co-IP and IB analysis of extracts of HEK293T cells transfected with FLAG-p62, along with HA-TRIM59 full-length WT or deletions (ΔR, ΔTM, B, and CC). ΔR, TRIM59 without RING domain; ΔTM, TRIM59 without the predicted transmembrane domain; B, B-box-type zinc finger domain; BafA1, bafilomycin A1; CC, coiled-coil domain; co-IP, co-immunoprecipitation; HA, hemagglutinin; IB, immunoblot; K63, lysine 63; Myc, avian myelocytomatosis virus oncogene cellular homolog; PDCD10, programmed cell death protein 10; p62, phosphotyrosine-independent ligand for the Lck SH2 domain of 62 kDa; Ub, ubiquitin; TRIM59, tripartite motif 59; WT, wild-type.
(TIF)

S1 Table Statistical analysis on the correlation between TRIM59 IHC scores and clinical parameters in patients with breast cancers. IHC, immunohistochemistry; TRIM59, tripartite motif 59.
(DOCX)

S2 Table Clinical features of breast cancer samples used in this study. (DOCX)

S3 Table Summary of yeast two-hybrid screening results for TRIM59-interacting proteins, classified in functional groups. TRIM59, tripartite motif 59.
(DOCX)

S1 Data Excel files containing the underlying numerical data for Figs 1A, 1B, 1D, 1E, 2B, 2D, 2E, 2F, 2G, 2H, 2I, 2J, 2K, 3B, 3D, 3G, 3I, 4A, 4H, 4I, 4L, 4M, 5D, 6B, 6C, 6D, 6J, 6K, 6M and S1A, S1B, S1C, S1D, S1E, S1F, S1G, S1H, S1I, S1B, and S1B. Data were listed in the indicated separate sheets.
(XLSX)

## Abbreviations

AMP: adenosine monophosphate
AMPK: AMP-activated protein kinase
B: B-box-type zinc finger domain
BafA1: bafilomycin A1
BRCA: breast invasive carcinoma
CC: coiled-coil domain
CCM: cerebral cavernous malformation
CDK: cyclin-dependent kinase
CK8: cytokeratin 8
co-IP: co-immunoprecipitation
CPTAC: Clinical Proteomic Tumor Analysis Consortium
CRISPR/Cas9: clustered regularly interspaced short palindromic repeats (CRISPR)-associated protein-9 nuclease
ER: estrogen receptor
ERK: extracellular signal–regulated kinase
ERM: Ezrin (Thr567)/Radixin (Thr564)/Moesin (Thr558)
FAT-H: focal adhesion targeting-homology
GBLP: guanine nucleotide-binding protein subunit beta-like protein
GFP: green fluorescent protein
GTPase: guanosine triphosphatase
HA: hemagglutinin
HDAC6: histone deacetylase 6
HE: hematoxylin–eosin
HEK: human embryonic kidney
IACUC: Institutional Animal Care and Use Committee
IB: immunoblot
IGF1R: type I insulin-like growth factor receptor
IgG: immunoglobulin G
IHC: immunohistochemistry
INPP4b: inositol polyphosphate-4-phosphatase type II b
IRES: internal ribosome entry site
JNK: c-Jun N-terminal kinase
KD: knockdown
KO: knockout
KRIT1: Krev/Rap1 Interacting Trapped 1
K63: lysine 63
LC3: microtubule-associated protein 1A/1B-light chain 3
MAP: mitogen-activated protein
MAPK: mitogen-activated protein kinase
MAT: mesenchymal-amoeboid transition
MLC: myosin light chain
Myc: avian myelocytomatosis virus oncogene cellular homolog
NBR1: neighbor of BRCA1 gene 1
NDP52: nuclear Domain 10 Protein 52
NIH: National Institute of Health
NIX: BCL2 interacting protein 3 like
NSG: NOD scid gamma
OE: overexpressing
OPTN: optineurin
OSM: osmosensing scaffold for mitogen-activated protein kinase kinase kinase-3
PDCD10: programmed cell death protein 10
p62: phosphotyrosine-independent ligand for the Lck SH2 domain of 62 kDa
qPCR: quantitative polymerase chain reaction
Rac1: Ras-related C3 botulinum toxin substrate 1
Ras: rat sarcoma
RING: really interesting new gene
RhoA: Ras homolog family member A
RNFT1: RING finger and transmembrane domain-containing protein 1
ROCK: Rho-associated coiled-coil kinase
RPPA: reverse phase protein array
sgRNA: single guide RNA
shRNA: short hairpin RNA
STK: Ste20-like kinase
TCGA: the Cancer Genome Atlas
TM: transmembrane domain
TRIM: tripartite motif
Ub: ubiquitin
UBA: Ub-associated domain
VEGFR2: vascular endothelial growth factor receptor 2
WCL: whole cell lysate
WT: wild-type
3-MA: 3-methyladenine
