# Supplementary figures and images for: TRIM59 promotes breast cancer motility by suppressing p62-selective autophagic degradation of PDCD10
Source: PLoS Biol. 2018 Nov 8;16(11):e3000051. doi: 10.1371/journal.pbio.3000051 (PMC6245796; doi:10.1371/journal.pbio.3000051)

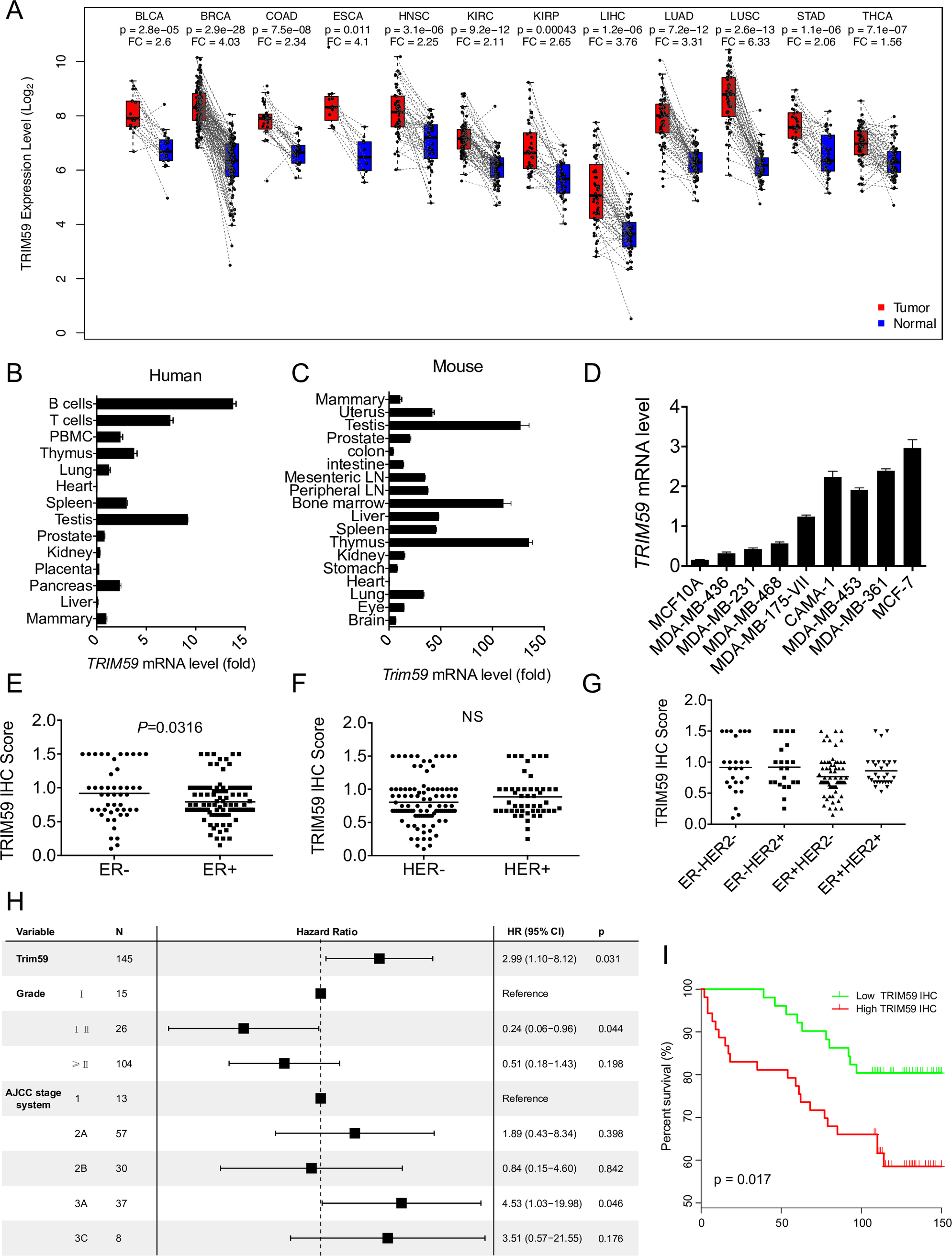

Supplement: S1 Fig — Related to Fig 1. (A) Fold change of TRIM59 expression across 12 cancer types compared with their paired controls (obtained from adjacent noncancerous tissues), based on TCGA dataset analysis. (B and C) mRNA expression profiles of TRIM59 in human (B) and mouse (C) tissues. (D) Quantitative RT-PCR of TRIM59 mRNA expression in human breast cancer cell lines. (E-G) TRIM59 IHC scores in subtypes of breast cancer based on HER and ER status. (H) Multivariate overall survival analysis of patient by using a Cox proportion hazards model. (I) Kaplan–Meier survival curves for Grade II or above breast cancer patients. Patients were divided into two groups according to TRIM59 staining scores (Low, with a score less than or equal to 0.75; High, with a score greater than 0.75). The underlying data can be found in S1 Data. ER, estrogen receptor; HER, human epidermal growth factor receptor 2; IHC, immunohistochemistry; RT-PCR, reverse transcription polymerase chain reaction; TCGA, the Cancer Genome Atlas; TRIM59, tripartite motif 59. (TIF) [file pbio.3000051.s001.tif]

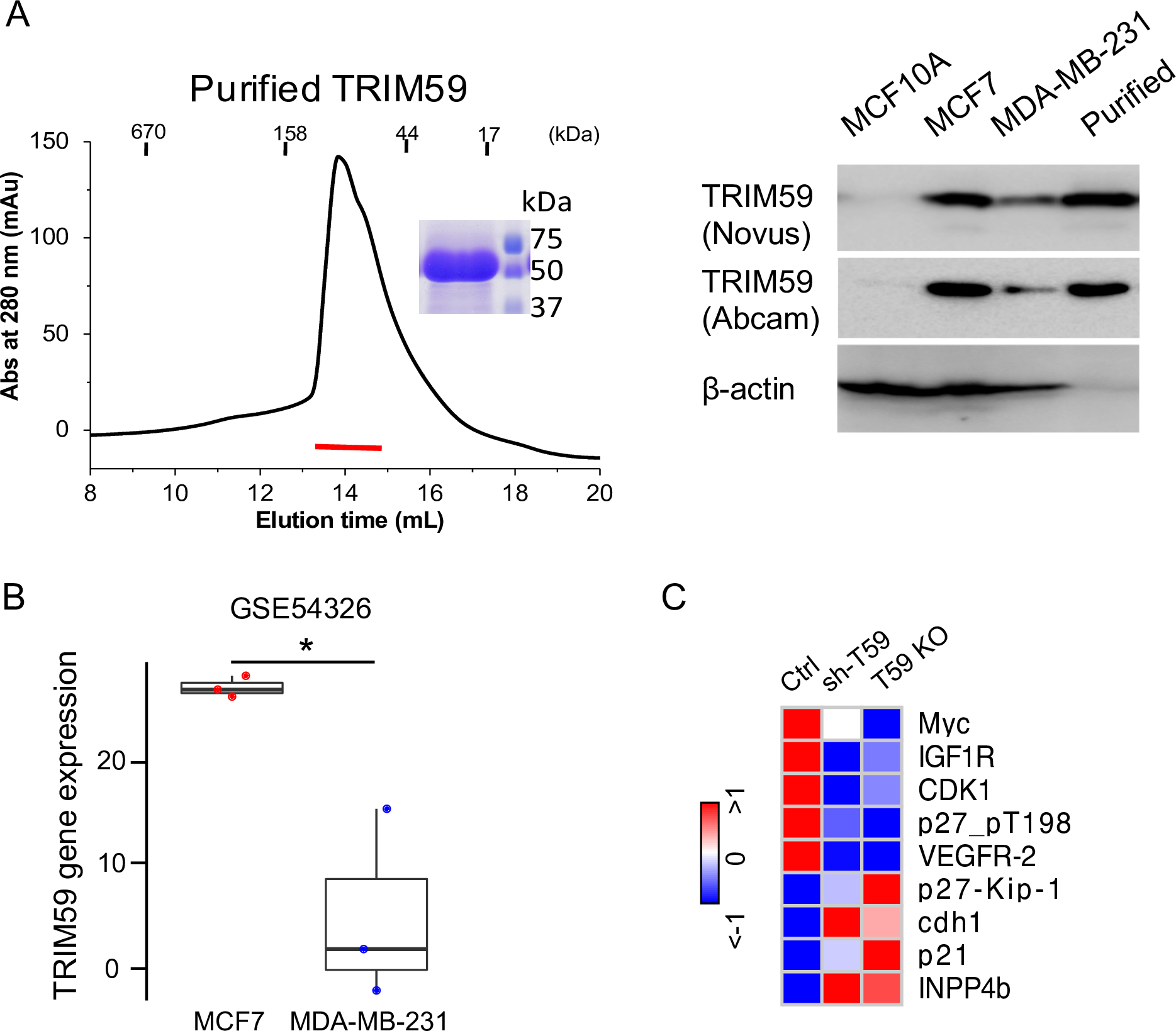

Supplement: S2 Fig — Related to Fig 3. (A) IB analysis of TRIM59 expression in MCF10A, MCF7, and MDA-MB-231 using two TRIM59 antibodies (Novus and Abcam). Purified TRIM59 protein was used as positive control (S2A, left). (B) TRIM59 mRNA expression levels in MCF7 and MDA-MB-231 cells revealed by RNA sequencing dataset analysis (GEO database: #GSE54326). n = 3. Data are presented as means ± SD. *P < 0.05. (C) RPPA analysis of alterations in protein expression levels or phosphorylation in Control, shTRIM59, and TRIM59 KO MCF7 cells. n = 3. The underlying data can be found in S1 Data. IB, immunoblot; RPPA, reverse phase protein array; TRIM59, tripartite motif 59. (TIF) [file pbio.3000051.s002.tif]

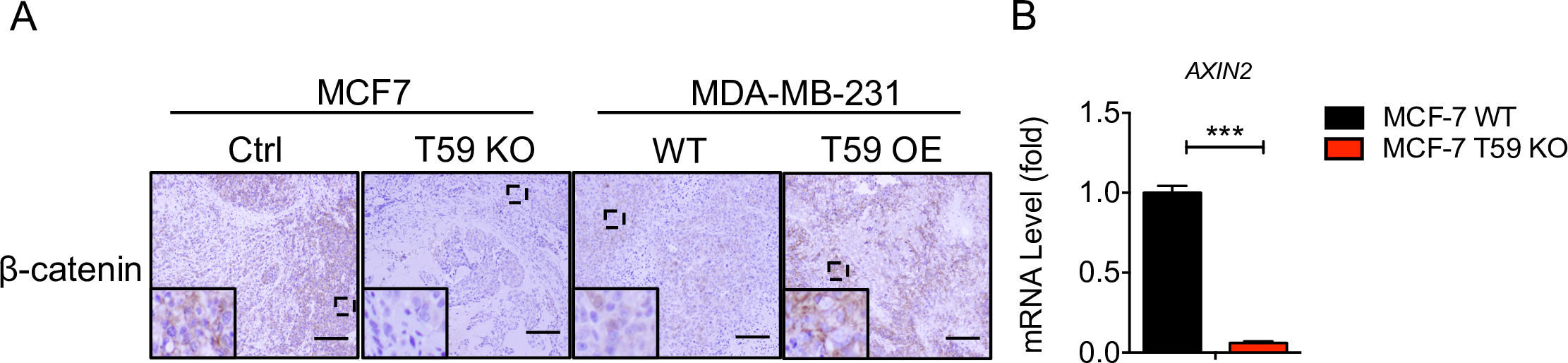

Supplement: S3 Fig — Related to Fig 4. (A) Representative immunochemistry staining of β-catenin in tissue sections from xenograft tumors of TRIM59 KO MCF7 cells or TRIM59 OE MDA-MB-231 cells compared with control cells. Scale bars: 200 μm. (B) qPCR quantification of the Wnt signaling pathway downstream gene AXIN2 in WT and TRIM59 KO MCF7 cells. n = 4. Data are presented as means ± SD. ***P < 0.001 versus WT. The underlying data can be found in S1 Data. KO, knockout; OE, overexpressed; qPCR, quantitative polymerase chain reaction; TRIM59, tripartite motif 59; WT, wild-type. (TIF) [file pbio.3000051.s003.tif]

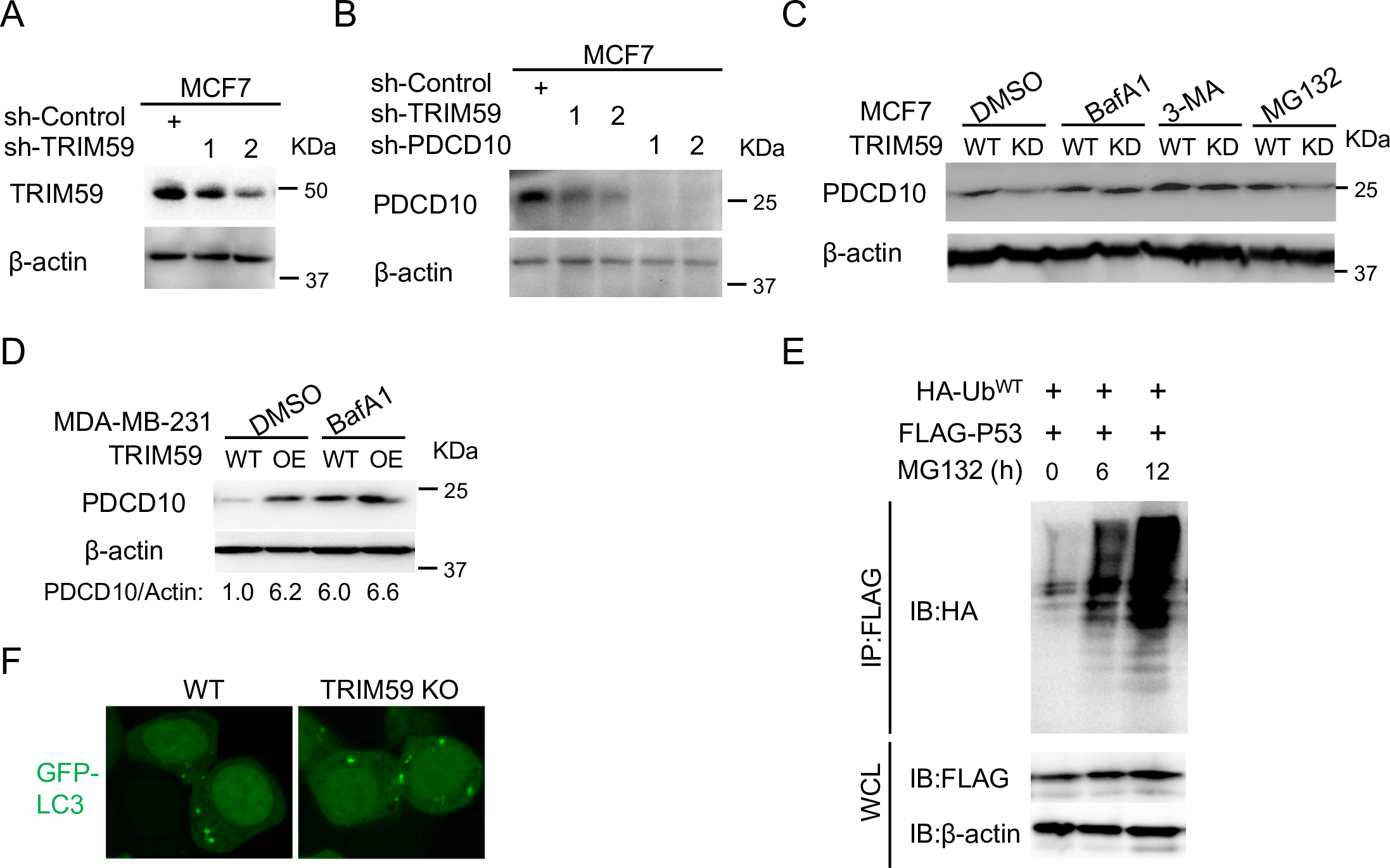

Supplement: S4 Fig — Related to Fig 5. (A) IB analysis of endogenous TRIM59 expression in MCF7 cells expressing two different shRNAs targeting TRIM59 compared with scramble shRNA (shControl). (B) IB analysis of endogenous PDCD10 expression in MCF7 cells expressing shTRIM59-1 or shTRIM59-2 compared with shControl. MCF7 cells expressing shPDCD10-1 or shPDCD10-2 targeted to PDCD10 were used as control. (C) IB analysis of PDCD10 in WT MCF7 or TRIM59 KD MCF7 cells treated with DMSO, BafA1, 3-MA, or MG132 for 6 hours. (D) IB analysis of PDCD10 protein levels in WT or TRIM59 OE MDA-MB-231 cells treated with DMSO or BafA1 for 12 hours. Quantitative comparisons of PDCD10 levels were analyzed by densitometric scanning of blots and normalized to β-actin. (E) Co-IP and IB analysis of HEK293T cells transfected with FLAG-p53, along with HA-tagged UbWT, followed by MG132 treatment (0, 6, or 12 hours). (F) Representative confocal images of WT or TRIM59 KO HEK293T cells transfected with GFP-LC3. BafA1, bafilomycin A1; co-IP, co-immunoprecipitation; GFP, green fluorescent protein; HA, hemagglutinin; IB, immunoblot; KD, knockdown; KO, knockout; LC3, microtubule-associated protein 1A/1B-light chain 3; OE, overexpressed; PDCD10, programmed cell death protein 10; shRNA, short hairpin RNA; TRIM59, tripartite motif 59; UbWT, ubiquitin WT; WT, wild-type; 3-MA, 3-methyladenine. (TIF) [file pbio.3000051.s004.tif]

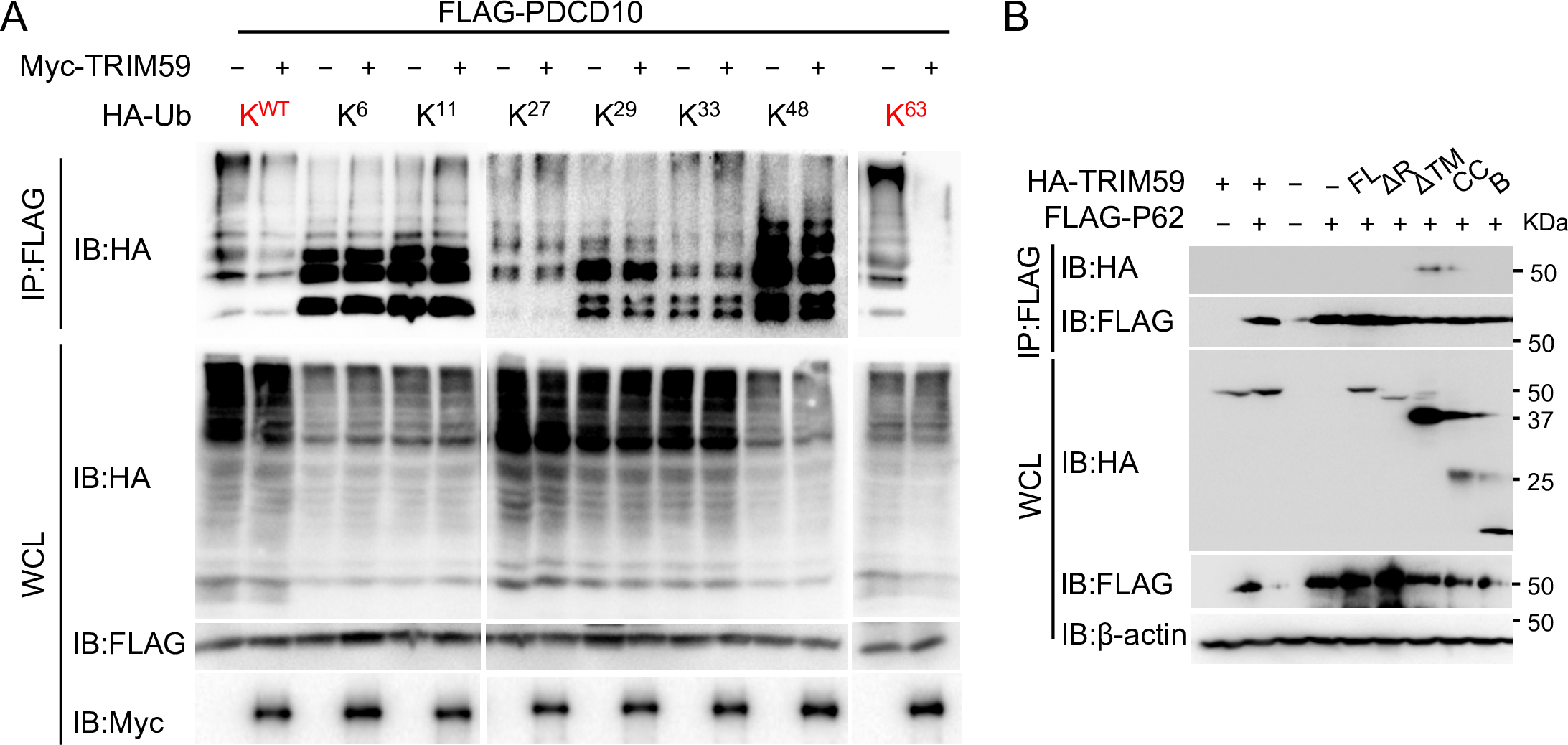

Supplement: S5 Fig — (A) HEK293T cells were transfected with FLAG-PDCD10 and HA-Ub (WT) or its mutants, along with Myc-TRIM59 or empty vector, in the presence of BafA1. Whole-cell lysates were immunoprecipitated with anti-FLAG beads and immunoblotted with indicated antibodies. (B) Co-IP and IB analysis of extracts of HEK293T cells transfected with FLAG-p62, along with HA-TRIM59 full-length WT or deletions (ΔR, ΔTM, B, and CC). ΔR, TRIM59 without RING domain; ΔTM, TRIM59 without the predicted transmembrane domain; B, B-box-type zinc finger domain; BafA1, bafilomycin A1; CC, coiled-coil domain; co-IP, co-immunoprecipitation; HA, hemagglutinin; IB, immunoblot; K63, lysine 63; Myc, avian myelocytomatosis virus oncogene cellular homolog; PDCD10, programmed cell death protein 10; p62, phosphotyrosine-independent ligand for the Lck SH2 domain of 62 kDa; Ub, ubiquitin; TRIM59, tripartite motif 59; WT, wild-type. (TIF) [file pbio.3000051.s005.tif]
