# Supplementary material for: TRIM59 promotes breast cancer motility by suppressing p62-selective autophagic degradation of PDCD10
Source: PLoS Biol. 2018 Nov 8;16(11):e3000051. doi: 10.1371/journal.pbio.3000051 (PMC6245796; doi:10.1371/journal.pbio.3000051)
Supplement: S1 Table — IHC, immunohistochemistry; TRIM59, tripartite motif 59. (DOCX) [file pbio.3000051.s006.docx]

| **S1 Table. Statistical analysis on the correlation between TRIM59 IHC scores and clinical parameters in patients with breast cancers.** | | | | |
| --- | --- | --- | --- | --- |
|  | TRIM59 IHC score | |  |  |
| Clinical Parameters | ≤ 0.75 | > 0.75 | χ^2^ | *P value* |
| Age |  |  | 14.86 | ＜0.001 |
| 55 or younger | 64 | 31 |  |  |
| Older than 55 | 21 | 38 |  |  |
| Tumor size (cm^3^) |  |  | 0.656 | 0.418 |
| ≤ 7 | 40 | 37 |  |  |
| > 7 | 45 | 32 |  |  |
| Pathological grade |  |  | 3.652 | 0.161 |
| Ⅰ+（Ⅰ-Ⅱ） | 29 | 15 |  |  |
| Ⅱ | 54 | 50 |  |  |
| （Ⅱ-Ⅲ）+Ⅲ | 2 | 4 |  |  |
| Tumor stage |  |  | 0.912 | 0.634 |
| T1 | 19 | 17 |  |  |
| T2 | 56 | 47 |  |  |
| T3 | 10 | 5 |  |  |
| AJCC stage (Version 6) |  |  | 0.417 | 0.812 |
| 1 | 6 | 7 |  |  |
| 2A+2B | 50 | 40 |  |  |
| 3A+3B+3C | 26 | 21 |  |  |
| positive lymph node |  |  | 3.938 | 0.14 |
| ≤ 15% | 40 | 39 |  |  |
| 15-50% | 31 | 15 |  |  |
| > 50% | 11 | 12 |  |  |
