# Supplementary material for: TRIM59 promotes breast cancer motility by suppressing p62-selective autophagic degradation of PDCD10
Source: PLoS Biol. 2018 Nov 8;16(11):e3000051. doi: 10.1371/journal.pbio.3000051 (PMC6245796; doi:10.1371/journal.pbio.3000051)
Supplement: S2 Table — (DOCX) [file pbio.3000051.s007.docx]

| **S2 Table. Clinical features of breast cancer samples used in this study.** | | | | | | | | | |
| --- | --- | --- | --- | --- | --- | --- | --- | --- | --- |
| Age | T | N | Pathological grade | Tumor size  (cm x cm x cm) | Lymph node number | Positive lymph  node number | AJCC stage  (V6) | Event  (0：Survival；  1：death) | Survival (month) |
| 48 | T2 | N0 | Ⅱ | 3.5×2.5×1 | 14 | 0 | 2A | 0 | 150 |
| 60 | T2 | N0 | Ⅱ | 4×3×3 | 12 | 0 | 2A | 0 | 150 |
| 45 | T2 | N0 | Ⅱ | 5×2.8×1.2 | 14 | 0 | 2A | 0 | 150 |
| 47 | T3 | N2 | Ⅰ-Ⅱ | 6×5×4 | 11 | 4 | 3A | 1 | 32 |
| 37 | T2 | N0 | Ⅱ | 2.5×2×2 | 13 | 0 | 2A | 0 | 149 |
| 54 | T3 | N1 | Ⅱ | 6×2.5×1.5 | 12 | 2 | 3A | 0 | 148 |
| 72 | T2 | N0 | Ⅰ-Ⅱ | 4×3×2 | 9 | 0 | 2A | 0 | 148 |
| 31 | T2 | N0 | Ⅰ-Ⅱ | 4.5×2.5×2 | 10 | 0 | 2A | 0 | 147 |
| 83 | T1 |  | Ⅰ | 2×2×1.5 |  |  |  | 1 | 145 |
| 50 | T3 | N3 | Ⅱ | 6×2.5×2 | 21 | 21 | 3C | 0 | 147 |
| 57 | T2 | N2 | Ⅰ-Ⅱ | 3.5×3×3 | 14 | 6 | 3A | 0 | 147 |
| 52 | T2 | N1 | Ⅰ | 3×2.5×2 | 6 | 1 | 2B | 0 | 147 |
| 76 | T2 | N0 | Ⅱ | 3×2×2 | 6 | 0 | 2A | 1 | 33 |
| 63 | T1 | N0 | Ⅱ | 2×1.5×1.5 | 10 | 0 | 1 | 0 | 146 |
| 49 | T2 | N0 | Ⅱ | 2.5×2×1.8 | 12 | 0 | 2A | 0 | 145 |
| 82 | T2 | N0 | Ⅱ | 3.4×3×3 | 11 | 0 | 2A | 1 | 54 |
| 48 | T3 | N2 | Ⅰ | 5.8×4×3 | 18 | 4 | 3A | 0 | 145 |
| 37 | T2 | N0 | Ⅱ-Ⅲ | 2.8×2.5×2.5 | 13 | 0 | 2A | 1 | 93 |
| 34 | T1 | N0 | Ⅱ | 2×2×1 | 9 | 0 | 1 | 0 | 145 |
| 73 | T2 | N2 | Ⅱ-Ⅲ | 2.5×1×1 | 8 | 7 | 3A | 1 | 114 |
| 59 | T1 | N0 | Ⅱ | 2×1.5×1 | 9 | 0 | 1 | 0 | 145 |
| 54 | T2 | N0 | Ⅱ | 3×3×3 | 7 | 0 | 2A | 1 | 97 |
| 56 | T2 | N0 | Ⅱ | 4×4×3 | 6 | 0 | 2A | 0 | 144 |
| 82 | T1 |  | Ⅱ | 1.5×1.5×1.5 |  |  |  | 0 | 143 |
| 72 | T2 | N2 | Ⅱ | 3×3×2 | 9 | 7 | 3A | 0 | 143 |
| 63 | T2 | N1 | Ⅰ-Ⅱ | 5×1.5×1.5 | 10 | 2 | 2B | 0 | 143 |
| 51 | T1 | N2 | Ⅱ | 2×2×1.5 | 1 | 1 | 3A | 1 | 18 |
| 44 | T3 | N1 | Ⅱ | 8×4×3 | 12 | 1 | 3A | 1 | 17 |
| 67 | T1 | N0 | Ⅰ-Ⅱ | 1.5×1×1 | 8 | 0 | 1 | 0 | 143 |
| 48 | T2 | N3 | Ⅱ | 5×4×3 | 11 | 10 | 3C | 1 | 11 |
| 47 | T1 | N0 | Ⅰ | 1×1×1 | 8 | 0 | 1 | 1 | 95 |
| 68 | T1 | N1 | Ⅰ | 2×1.5×1 | 10 | 2 | 2A | 1 | 82 |
| 55 | T2 | N2 | Ⅱ | 4×3×3 | 8 | 4 | 3A | 0 | 142 |
| 76 | T3 |  | Ⅰ | 7×5×3 |  |  |  | 0 | 141 |
| 72 | T1 | N1 | Ⅰ-Ⅱ | 1.2×1×0.8 | 12 | 1 | 2A | 0 | 141 |
| 45 | T2 | N0 | Ⅰ | 3×3×2 | 6 | 0 | 2A | 0 | 141 |
| 44 | T2 | N0 | Ⅰ-Ⅱ | 1×0.5×0.5 | 9 | 0 | 2A | 0 | 141 |
| 77 | T2 | N2 | Ⅱ | 3×3×2.5 | 14 | 6 | 3A | 0 | 141 |
| 60 | T2 | N2 | Ⅰ-Ⅱ | 3.5×2.5×3 | 12 | 8 | 3A | 0 | 141 |
| 70 | T2 | N0 | Ⅱ | 2.5×2×0.5 | 7 | 0 | 2A | 1 | 39 |
| 44 | T2 | N1 | Ⅱ | 5×4×2.5 | 16 | 3 | 2B | 0 | 140 |
| 38 | T2 | N2 | Ⅰ-Ⅱ | 3.5×3×2 | 8 | 5 | 3A | 0 | 140 |
| 57 | T1 | N1 | Ⅰ-Ⅱ | 2×2×1.5 | 11 | 1 | 2A | 0 | 139 |
| 64 | T2 | N1 | Ⅱ | 3×3×2 | 6 | 2 | 2B | 0 | 138 |
| 44 | T3 | N2 | Ⅱ | 7×6×6 | 7 | 5 | 3A | 0 | 138 |
| 48 | T2 | N0 | Ⅰ | 2×3×0.7 | 14 | 0 | 2A | 1 | 32 |
| 47 | T2 | N0 | Ⅰ | 1.5×3×1 | 7 | 0 | 2A | 0 | 138 |
| 47 | T1 | N1 | Ⅰ | 2×1.5×1 | 9 | 2 | 2A | 0 | 137 |
| 33 | T3 | N3 | Ⅱ | 10×10×4 | 14 | 11 | 3C | 0 | 137 |
| 46 | T2 | N2 | Ⅱ | 5×5×5 | 7 | 6 | 3A | 1 | 7 |
| 39 | T2 | N3 | Ⅰ | 3×3×2 | 17 | 14 | 3C | 0 | 137 |
| 46 | T2 | N0 | Ⅱ | 5×3.5×2.5 | 10 | 0 | 2A | 0 | 137 |
| 40 | T1 | N2 | Ⅱ | 2×2×0.6 | 7 | 4 | 3A | 0 | 137 |
| 61 | T2 | N0 | Ⅱ | 4.5×2×2 | 10 | 0 | 2A | 0 | 136 |
| 61 | T1 | N1 | Ⅰ | 2×2×1 | 8 | 3 | 2A | 0 | 136 |
| 48 | T2 | N2 | Ⅱ | 2.5×1.5×1 | 10 | 6 | 3A | 1 | 125 |
| 40 | T2 | N1 | Ⅰ | 3×3×2 | 14 | 2 | 2B | 1 | 23 |
| 62 | T2 | N1 | Ⅰ-Ⅱ | 3×2.5×3 | 14 | 2 | 2B | 0 | 135 |
| 36 | T1 | N2 | Ⅱ | 2×1×1 | 10 | 5 | 3A | 0 | 135 |
| 38 | T3 | N1 | Ⅰ-Ⅱ | 6×2×2 | 9 | 2 | 3A | 0 | 135 |
| 73 | T2 | N1 | Ⅰ-Ⅱ | 2.5×2×1.5 | 9 | 1 | 2B | 0 | 135 |
| 45 | T1 | N1 | Ⅰ-Ⅱ | 2×1×1 | 13 | 1 | 2A | 1 | 47 |
| 76 | T2 | N0 | Ⅱ | 3×3×2 | 12 | 0 | 2A | 1 | 77 |
| 47 | T2 | N1 | Ⅱ | 3×2×1.5 | 10 | 3 | 2B | 0 | 134 |
| 61 | T2 | N1 | Ⅱ | 2.5×2×2 | 14 | 1 | 2B | 0 | 134 |
| 78 | T2 | N0 | Ⅱ | 4×4×3 | 14 | 0 | 2A | 1 | 92 |
| 55 | T2 | N1 | Ⅰ | 2.5×2×1.2 | 11 | 2 | 2B | 1 | 31 |
| 66 | T2 | N0 | Ⅱ | 3×2×1.5 | 12 | 0 | 2A | 0 | 133 |
| 52 | T2 | N1 | Ⅰ | 4×3.5×2 | 7 | 2 | 2B | 0 | 133 |
| 48 | T2 | N0 | Ⅰ-Ⅱ | 4.5×3.5×2.5 | 11 | 0 | 2A | 0 | 132 |
| 41 | T1 | N0 | Ⅱ | 2×2×1.5 | 17 | 0 | 1 | 0 | 132 |
| 51 | T2 | N0 | Ⅱ | 3×3×2 | 10 | 0 | 2A | 0 | 132 |
| 45 | T2 | N0 | Ⅱ | 3×2×2 | 7 | 0 | 2A | 0 | 132 |
| 75 | T2 | N2 | Ⅱ-Ⅲ | 3.5×2.5×2 | 10 | 7 | 3A | 1 | 53 |
| 47 | T2 | N0 | Ⅱ | 4×3×2.5 | 14 | 0 | 2A | 0 | 131 |
| 56 | T2 | N1 | Ⅱ | 3.5×3×2.5 | 13 | 1 | 2B | 0 | 131 |
| 74 | T3 | N2 | Ⅰ-Ⅱ | 7×6×3 | 10 | 4 | 3A | 1 | 23 |
| 32 | T1 | N1 | Ⅱ | 1.5×1×0.8 | 14 | 1 | 2A | 0 | 130 |
| 50 | T1 | N2 | Ⅰ-Ⅱ | 1×1×1 | 11 | 4 | 3A | 0 | 130 |
| 72 | T2 | N1 | Ⅱ | 3×2×1 | 8 | 3 | 2B | 0 | 130 |
| 36 | T2 | N3 | Ⅱ | 3×2×1 | 20 | 13 | 3C | 1 | 15 |
| 46 | T2 | N1 | Ⅰ | 3×3×1 | 11 | 3 | 2B | 0 | 129 |
| 54 | T2 | N0 | Ⅱ | 4.5×3×1.5 | 16 | 0 | 2A | 1 | 60 |
| 63 | T2 | N1 | Ⅱ-Ⅲ | 4.5×3×1 | 17 | 2 | 2B | 1 | 110 |
| 54 | T1 | N1 | Ⅰ | 2×2×2 | 7 | 1 | 2A | 0 | 129 |
| 50 | T2 | N1 | Ⅱ | 3×3×2.5 | 13 | 3 | 2B | 0 | 128 |
| 53 | T2 | N2 | Ⅱ | 3×2.5×2 | 8 | 4 | 3A | 1 | 63 |
| 63 | T1 | N1 | Ⅱ | 2×1.5×1 | 7 | 2 | 2A | 1 | 35 |
| 32 | T2 | N1 | Ⅱ | 4×3×2 | 11 | 3 | 2B | 0 | 128 |
| 37 | T1 | N3 | Ⅱ | 1×1×1 | 30 | 26 | 3C | 0 | 128 |
| 81 | T2 |  | Ⅱ | 3×2×1.5 |  |  |  | 0 | 128 |
| 55 | T2 | N1 | Ⅱ | 3×2×1.5 | 10 | 3 | 2B | 0 | 128 |
| 40 | T2 | N0 | Ⅰ-Ⅱ | 5×4.5×4 | 11 | 0 | 2A | 1 | 44 |
| 29 | T2 | N2 | Ⅱ | 3×3×2 | 9 | 9 | 3A | 1 | 78 |
| 32 | T2 | N1 | Ⅱ | 2×3.2×4.2 | 15 | 2 | 2B | 0 | 128 |
| 45 | T2 | N1 | Ⅱ | 5×3×1 | 8 | 1 | 2B | 0 | 127 |
| 31 | T3 | N2 | Ⅱ | 15×15×7 | 8 | 8 | 3A | 1 | 4 |
| 66 | T2 | N0 | Ⅰ-Ⅱ | 3×3×2 | 8 | 0 | 2A | 0 | 127 |
| 54 | T2 | N2 | Ⅲ | 4.5×2.5×2 |  |  | 3A | 1 | 4 |
| 52 | T2 | N2 | Ⅱ | 4×3×0.5 | 14 | 6 | 3A | 0 | 127 |
| 48 | T2 | N1 | Ⅱ | 2.5×2×1.5 | 11 | 2 | 2B | 0 | 126 |
| 71 | T2 | N0 | Ⅱ | 2.5×2.5×2.2 | 12 | 0 | 2A | 1 | 62 |
| 49 | T2 | N0 | Ⅱ | 3×3×1.5 | 13 | 0 | 2A | 0 | 120 |
| 69 | T2 | N1 | Ⅱ | 2.5×2×2 | 8 | 3 | 2B | 0 | 120 |
| 46 | T1 | N1 | Ⅱ | 2×1×1 | 10 | 2 | 2A | 0 | 119 |
| 49 | T2 | N0 | Ⅱ | 3×3×2 | 10 | 0 | 2A | 0 | 119 |
| 82 | T3 | N2 | Ⅱ | 6×4×1.5 |  |  | 3A | 1 | 2 |
| 52 | T2 | N2 | Ⅱ | 2.5×2×1.5 | 12 | 8 | 3A | 1 | 110 |
| 44 | T2 | N2 | Ⅱ | 3.5×1.5×1 | 15 | 4 | 3A | 0 | 118 |
| 51 | T2 | N0 | Ⅱ | 2.5×2×1.5 | 5 | 0 | 2A | 0 | 118 |
| 43 | T3 | N2 | Ⅱ | 7×7×6 | 21 | 8 | 3A | 0 | 117 |
| 67 | T2 | N1 | Ⅱ | 3×3×2 | 12 | 1 | 2B | 0 | 117 |
| 37 | T1 | N0 | Ⅱ-Ⅲ | 1.5×1.5×1.5 | 5 | 0 | 1 | 1 | 85 |
| 51 | T1 | N1 | Ⅰ | 1.8×1.5×1 | 16 | 3 | 2A | 0 | 116 |
| 49 | T2 | N0 | Ⅰ-Ⅱ | 3×2.5×2 | 27 | 0 | 2A | 0 | 116 |
| 51 | T2 | N0 | Ⅰ-Ⅱ | 4×2×2 | 10 | 0 | 2A | 0 | 115 |
| 64 | T2 | N0 | Ⅱ | 3×3×3 | 5 | 0 | 2A | 1 | 68 |
| 52 | T2 | N3 | Ⅱ | 5×5×2 | 12 | 12 | 3C | 1 | 61 |
| 68 | T2 | N1 | Ⅱ | 3×2.5×2 | 13 | 1 | 2B | 0 | 115 |
| 39 | T2 | N2 | Ⅱ | 3×2×1.5 | 18 | 5 | 3A | 0 | 114 |
| 47 | T1 | N2 | Ⅰ-Ⅱ | 2×2×2 | 15 | 6 | 3A | 0 | 114 |
| 41 | T2 | N1 | Ⅰ-Ⅱ | 3.8×2.5×1 | 13 | 1 | 2B | 0 | 114 |
| 76 | T1 | N0 | Ⅰ-Ⅱ | 1.5×1.5×1.5 | 12 | 0 | 1 | 0 | 113 |
| 61 | T2 | N0 | Ⅱ | 2.5×2.5×2.5 | 14 | 0 | 2A | 0 | 113 |
| 65 | T2 | N0 | Ⅱ | 3×2.5×1.5 | 10 | 0 | 2A | 0 | 112 |
| 60 | T2 | N0 | Ⅱ | 2.5×2.5×2 | 19 | 0 | 2A | 0 | 112 |
| 50 | T2 | N2 | Ⅱ | 5×3×2.5 | 11 | 5 | 3A | 0 | 112 |
| 42 | T2 | N1 | Ⅱ | 5×5×4 | 11 | 1 | 2B | 0 | 112 |
| 40 | T2 | N0 | Ⅱ | 5×5×2 | 2 | 0 | 2A | 0 | 112 |
| 40 | T2 | N0 | Ⅱ | 4×2.5×2 | 18 | 0 | 2A | 0 | 112 |
| 57 | T1 | N0 | Ⅰ-Ⅱ | 2×2×2 | 11 | 0 | 1 | 0 | 111 |
| 47 | T2 | N0 | Ⅱ | 2.2×1.5×1.5 | 16 | 0 | 2A | 0 | 111 |
| 67 | T2 | N1 | Ⅱ | 4×3×3 | 8 | 2 | 2B | 0 | 111 |
| 59 | T2 | N0 | Ⅱ | 3.5×2×2 | 15 | 0 | 2A | 0 | 111 |
| 46 | T2 | N2 | Ⅱ | 3.5×3×2 | 10 | 4 | 3A | 0 | 111 |
| 34 | T2 | N1 | Ⅱ | 3×2.5×2 | 17 | 3 | 2B | 0 | 110 |
| 73 | T1 | N2 | Ⅱ | 2×2×2 | 14 | 4 | 3A | 1 | 79 |
| 56 | T2 | N2 | Ⅱ | 4×3.5×3 | 9 | 5 | 3A | 1 | 80 |
| 59 | T1 | N0 | Ⅱ | 0.5×0.5×0.5 | 14 | 0 | 1 | 0 | 110 |
| 53 | T1 | N0 | Ⅱ | 1.5×1.5×1 | 11 | 0 | 1 | 0 | 110 |
| 72 | T1 | N1 | Ⅱ | 2×1.5×1 | 10 | 1 | 2A | 1 | 46 |
| 52 | T2 | N1 | Ⅱ | 3×3×3 | 11 | 2 | 2B | 0 | 109 |
| 75 | T2 | N1 | Ⅱ | 3.5×3×1 | 13 | 2 | 2B | 1 | 59 |
| 33 | T2 | N3 | Ⅱ | 3×3×2 | 16 | 12 | 3C | 0 | 109 |
| 64 | T2 | N0 | Ⅱ | 2.5×2×1 | 8 | 0 | 2A | 0 | 109 |
| 43 | T2 | N2 | Ⅱ | 3×3×1.5 | 16 | 6 | 3A | 0 | 109 |
| 71 | T1 | N0 | Ⅱ | 2×2×2 | 11 | 0 | 1 | 0 | 109 |
| 49 | T1 | N1 | Ⅱ | 2×2×2 | 10 | 2 | 2A | 0 | 108 |
| 83 | T2 | N2 | Ⅱ | 3.5×3×1 | 12 | 5 | 3A | 0 | 108 |
| 50 | T1 | N0 | Ⅱ | 1.5×1.5×1 | 10 | 0 | 1 | 0 | 108 |
| 54 | T3 | N2 | Ⅱ | 10×8×5 | 13 | 9 | 3A | 1 | 9 |
| 42 | T3 | N0 | Ⅱ | 5.5×4×3.5 | 16 | 0 | 2B | 0 | 108 |
| 42 | T1 | N1 | Ⅱ | 2×2×1.5 | 12 | 1 | 2A | 0 | 108 |
| 51 | T2 | N0 | Ⅱ | 4×3×3 | 12 | 0 | 2A | 0 | 107 |
