# Supplementary material for: TRIM59 promotes breast cancer motility by suppressing p62-selective autophagic degradation of PDCD10
Source: PLoS Biol. 2018 Nov 8;16(11):e3000051. doi: 10.1371/journal.pbio.3000051 (PMC6245796; doi:10.1371/journal.pbio.3000051)
Supplement: S3 Table — TRIM59, tripartite motif 59. (DOCX) [file pbio.3000051.s008.docx]

**S3 Table. Summary of yeast-two hybrid screening results for TRIM59-interacting proteins, classified in functional groups.**

| **Gene Symbol** | **Protein name** | **Function Annotation** |
| --- | --- | --- |
| **PDCD10** | Programmed cell death protein 10 | Apoptosis; this study |
| SPTSSA | Serine palmitoyltransferase small subunit A | Transcription factor |
| TEX30 | Testis-expressed sequence 30 protein |  |
| **RNFT1** | RING finger and transmembrane domain-containing protein 1 | A putative E3 ligase |
| CRIPT | Cysteine-rich PDZ-binding protein | Scaffold protein binding |
| FDPS | Farnesyl pyrophosphate synthase | Acyltransferase |
| DIEXF | Digestive organ expansion factor homolog isoform X1 | rRNA binding |
| QRICH1 | Glutamine-rich protein 1 | Transcription factor |
| PBX1 | Pre B cell leukemia homeobox 1 | DNA-directed RNA polymerase |
| LARP4B | La ribonucleoprotein domain family, member 4B | Ribonucleoprotein |
| MPP5 | Membrane protein, palmitoylated 5 | Tight junction |
| SLC35F2 | Solute carrier family 35, member F2 |  |
